# β-Phase Yb_5_Sb_3_H_*x*_: Magnetic and Thermoelectric Properties Traversing from an Electride to a Semiconductor

**DOI:** 10.1021/acs.inorgchem.4c00254

**Published:** 2024-04-23

**Authors:** Ashlee K. Hauble, Tanner Q. Kimberly, Kamil M. Ciesielski, Nicholas Mrachek, Maxwell G. Wright, Valentin Taufour, Ping Yu, Eric S. Toberer, Susan M. Kauzlarich

**Affiliations:** † Department of Chemistry, University of California, One Shields Ave, Davis, California 95616, United States; ‡ Department of Physics, Colorado School of Mines, 1500 Illinois St, Golden, Colorado 80401, United States; § Department of Physics and Astronomy, University of California, One Shields Ave, Davis, California 95616, United States; ∥ Nuclear Magnetic Resonance Facility, University of California, One Shields Ave, Davis, California 95616, United States

## Abstract

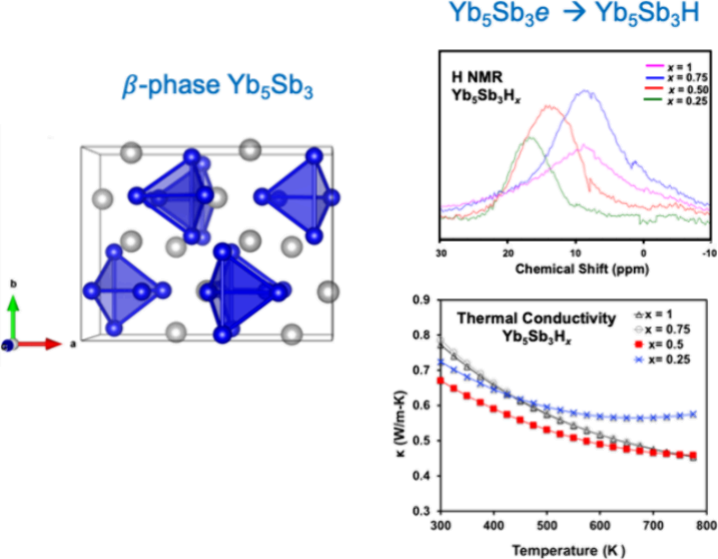

An electride is a compound that contains a localized electron in an empty crystallographic site. This class of materials has a wide range of applications, including superconductivity, batteries, photonics, and catalysis. Both polymorphs of Yb_5_Sb_3_ (the orthorhombic Ca_5_Sb_3_F structure type (β phase) and hexagonal Mn_5_Si_3_ structure type (α phase)) are known to be electrides with electrons localized in 0D tetrahedral cavities and 1D octahedral chains, respectively. In the case of the orthorhombic β phase, an interstitial H can occupy the 0D tetrahedral cavity, accepting the anionic electron that would otherwise occupy the site, providing the formula of Yb_5_Sb_3_H_*x*_. DFT computations show that the hexagonal structure is energetically favored without hydrogen and that the orthorhombic structure is more stable with hydrogen. Polycrystalline samples of orthorhombic β phase Yb_5_Sb_3_H_*x*_ (*x* = 0.25, 0.50, 0.75, 1.0) were synthesized, and both PXRD lattice parameters and ^1^H MAS NMR were used to characterize H composition. Magnetic and electronic transport properties were measured to characterize the transition from the electride (semimetal) to the semiconductor. Magnetic susceptibility measurements indicate a magnetic moment that can be interpreted as resulting from either the localized antiferromagnetically coupled electride or the presence of a small amount of Yb^3+^. At lower H content (*x* = 0.25, 0.50), a low charge carrier mobility consistent with localized electride states is observed. In contrast, at higher H content (*x* = 0.75, 1.0), a high charge carrier mobility is consistent with free electrons in a semiconductor. All compositions show low thermal conductivity, suggesting a potentially promising thermoelectric material if charge carrier concentration can be fine-tuned. This work provides an understanding of the structure and electronic properties of the electride and semiconductor, Yb_5_Sb_3_H_*x*_, and opens the door to the interstitial design of electrides to tune thermoelectric properties.

## Abstract 2

The β-phase of Yb_5_Sb_3_ in transition from an electride to a hydride is studied. ^1^H NMR shows a shift consistent with the shorter Yb−H distances indicated by the decreasing *a* lattice parameter, and all samples exhibit ultralow thermal conductivity, an important property for potential thermoelectric materials.

## Introduction

Electrides contain an anionic electron trapped in a vacant crystallographic site independent of any atom in the lattice. In many cases, interactions between the anionic electrons and the cationic host contribute to the structural stability of the compound.^1^ Electrides were originally discovered in organic salts in the 1980s,^2^ and in the last 20 years, this class of materials has been expanded to include a variety of inorganic compounds with different dimensions of confined electron space (0D cavities, 1D chains, 2D sheets, and 3D structures).^3^ Given that functional properties of electrides are closely linked to the topology of the confined electrons, this structural diversity has generated great interest in electrides for applications such as superconductivity, catalysis, batteries, spintronics, metal–insulator transitions, nonlinear optics, and photonics.^1,3^ However, the study of electrides is still in its infancy, and as they are better understood, these materials are likely to find applications in a variety of other fields.^1^

Exploration of new electrides via computational screening is becoming increasingly common using a crystal structure prediction tool^3^ since all known electrides contain an excess of highly electropositive cations and the localization of electride states is more extreme when low-dimensional cavities are also present.^4^ The two “polymorphs” of Yb_5_Sb_3_ fit this description, as the orthorhombic (*Pnma*) β-Yb_5_Sb_3_ structure type or Ca_5_Sb_3_F structure type contains 0D isolated cavities within Yb_4_ tetrahedra, and the hexagonal (*P6*_*3*_*/mmc*) Mn_5_Si_3_ or α-Yb_5_Sb_3_ structure type contains 1D chains of interstitial cavities surrounded by 6Yb cations.^4,5^ With 5 Yb^2+^ ions and 3 Sb^3–^ ions, the composition can be written as (Yb_5_Sb_3_^+^)(*e*^–^),^6−8^ and recent theoretical studies have confirmed that they are electrides.^4,5^

**Figure 1:**
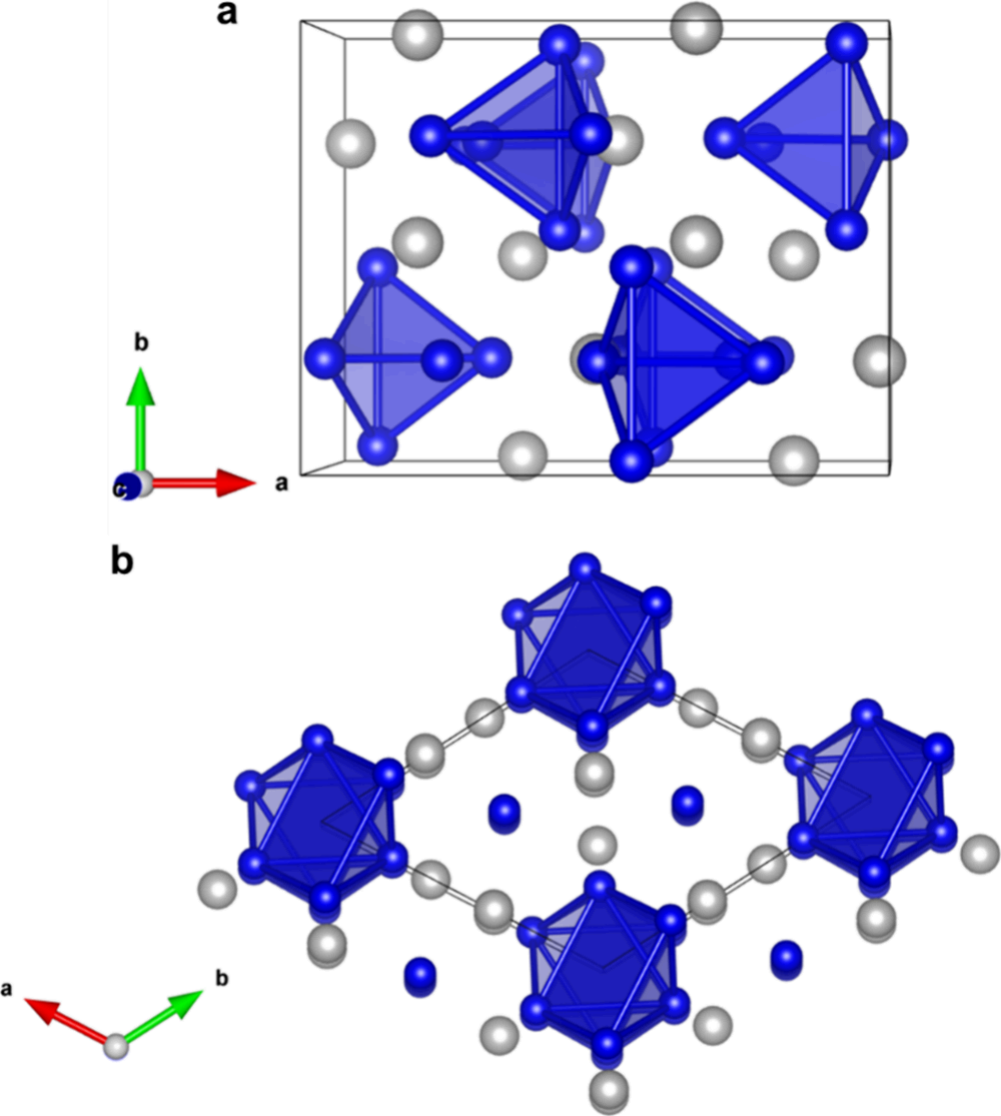
Structure of the (a) orthorhombic β- and (b) hexagonal α-structure types of Yb_5_Sb_3_H_*x*_. Yb and Sb atoms are colored blue and gray, respectively. The polyhedra show the interstitial 0D and 1D cavities that house either an anionic electron or an H^–^. H atom is not shown.

Both structure types are known to host interstitial impurities that can accept the extra electron, making them highly tunable,^6,7^ and it has been shown that the orthorhombic β-Yb_5_Sb_3_ is only stable when an interstitial H or F atom is present.^7^ Given this information, the β-Yb_5_Sb_3_ phase is better described according to composition as Yb_5_Sb_3_H_*x*_, and the structure type was reclassified as Ca_5_Sb_3_F. This finding went unknown for many years due to the presence of H as an impurity in commercial alkaline and rare earth metals and the experimental difficulty in identifying H atoms.^8^ In this orthorhombic structure type, four interstitial cavities in the unit cell accommodate either a localized anionic electron or a H (or F) atom. When the interstitial site is fully occupied, electron counting suggests that Yb_5_Sb_3_H should be a charge-balanced semiconductor rather than an electride. It is unclear how much H is required to drive the transition from the Mn_5_Si_3_ structure type to the β-Yb_5_Sb_3_ type, or how the electronic properties change as H content is increased for *x* = 0 to 1.0.^5,7,8^ A theoretical study on Yb_5_Sb_3_, Yb_5_Sb_3_H, and Yb_5_Sb_3_F showed the presence of four electride bands, one for each anionic electron in the unit cell, in the hypothetical orthorhombic Yb_5_Sb_3_ (the polyhedra shown in Figure 1) that are not present in the charge-balanced Yb_5_Sb_3_H or Yb_5_Sb_3_F structures, supporting the idea that H and F act as electron acceptors and occupy the cavities where the confined electrons would be located.^5^ Varying F content in Yb_5_Sb_3_F_*x*_ (*x* = 0, 0.25, 0.50, 0.75, 1.0) caused the electride bands to disappear one at a time, suggesting that the electronic properties can be tuned from an electride to a semiconductor/insulator by varying F or H content, making this material interesting for a wide variety of applications.

The anionic electrons present in electrides are free from onsite electron–nuclear interactions since they are not bound to a particular atom,^5^ which can lead to unique properties like high charge carrier mobility,^9^ anisotropic electronic properties,^1^ low-dimensional electronic structures, and high electron concentration,^3,10,11^ all qualities that are desirable for thermoelectric applications.^12−15^ Many ytterbium antimonides have been studied for thermoelectric applications due to their high thermal stability and tunability,^16−20^ but neither Yb_5_Sb_3_ nor Yb_5_Sb_3_H_*x*_ has been investigated, despite a high Seebeck coefficient reported for the orthorhombic phase and low electrical resistivity published for the hexagonal phase.^21−23^ Additionally, research on Zintl hydrides and other intermetallics shows that incorporation of small amounts of H can dramatically change the chemical structure and physical properties.^24−26^ The rich structural chemistry of Yb_5_Sb_3_ and Yb_5_Sb_3_H_*x*_ has been widely explored,^4−8,27−30^ but the physical and electronic properties have not been well characterized. Here, we show that the H content can be tuned to control the chemical and physical properties in orthorhombic Yb_5_Sb_3_H_*x*_ and apply thermoelectric measurements to interrogate the electronic properties across the transition from an electride to a charge-balanced semiconductor.

## Methods

### Computational Methods

First-principles calculations were carried out in the framework of density functional theory (DFT) as implemented in the Quantum ESPRESSO code.^31,32^ The exchange-correlation potential was described by the generalized gradient approximation (GGA) in the Perdew–Burke–Ernzerhof (PBE) functional.^33,34^ The projector-augmented wave (PAW) method was applied to electron–core interactions.^35^ Plane waves with a kinetic energy cutoff of 680 eV were used as the basis set, and a 3 × 4 × 4 and 3 × 3 × 4 k-point mesh was used for the first Brillouin zone sampling for the orthorhombic and hexagonal structures, respectively. Lattice structure relaxations were performed such that all residual forces on the atoms were smaller than 0.01 eV/Å. Beginning with the experimental crystallographic coordinates of the orthorhombic and hexagonal structures,^21,22^ the hydrogenated structures were computationally constructed by adding hydrogen atoms to the interstitial crystallographic sites. Structure relaxations were carried out for *x* = 0.0, 0.25, 0.50, 0.75, and 1.0 and *x* = 0.0, 0.50, and 1.0 for the Yb_5_Sb_3_H_*x*_ orthorhombic and hexagonal phases, respectively. Calculations on the hexagonal and orthorhombic phases were performed in the antiferromagnetic configuration. Structure relaxations were also done on the elements Yb and Sb using experimental crystallographic coordinates.^36,37^ The total energy of H was calculated by using one isolated hydrogen with a vacuum space of 8 Å^3^ to exclude the interaction of neighboring images due to periodic boundary conditions. Formation energies were computed as described below. The final atomic positions for the relaxed structures are provided in the Supporting Information (SI), Table S1.

### Synthesis

Polycrystalline synthesis of Yb_5_Sb_3_H_*x*_ (*x* = 0.25, 0.50, 0.75, and 1.0) was carried out via ball milling the binary precursor Yb_4_Sb_3_ with stoichiometric amounts of Yb metal (Stanford Materials, 99.99%) and YbH_2_ (American Elements, 99.999%) and annealing the milled powder. Yb_4_Sb_3_ was used instead of the elements as a way to control H content since rare earths are known to contain H impurities^38^ and Yb_4_Sb_3_ is not known to take up H. The binary precursor, Yb metal and YbH_2_, were combined in a 65 mL stainless steel ball mill vial with two 12.7 mm diameter stainless steel balls in an Ar-filled glovebox. The vial was then sealed in a polyethylene bag and milled in a SPEX 8000 M ball mill for 30 min, transferred back into the glovebox to be scraped with a stainless-steel spatula, and then milled for an additional 30 min. The resulting powder (∼5 g) was transferred into a Ta tube (7 cm length, 1 cm OD) which was crimped and welded using an arc welder in an Ar atmosphere. The Ta tube was flame-sealed in an evacuated (<50 mTorr) quartz ampule and annealed in a box furnace at 800 °C for 7 days (heating rate 200 °C/h). Powder X-ray diffraction analysis confirmed that orthorhombic Yb_5_Sb_3_H_*x*_ was synthesized and *x* is nominal based on the reaction coefficients (Rietveld refinement and statistics in Figure S1 and Table S2).

The Yb_4_Sb_3_ precursor was synthesized from stoichiometric amounts of cut Yb pieces (Stanford Materials, 99.99%) and Sb (shot, 5NPlus, 99.999%) that were ground to a powder in an agate mortar and pestle and ball-milled using the same milling scheme described above. The powder was then sealed in a Nb tube and quartz jacket and annealed in a box furnace at 600 °C for 12 h (heating rate 100 °C/h). The purity of the sample was confirmed by powder X-ray diffraction (PXRD) prior to use (Rietveld refinement and statistics in Figure S2 and Table S3).

No uncommon hazards are noted.

### Spark Plasma Sintering

An agate mortar and pestle were used to grind annealed powders. The powder (∼2.5–3 g) was sieved and packed into a 12.7 mm ID graphite die (lined with graphite foil) inside an Ar-filled glovebox and placed into the chamber of a Fuji Electronic Industrial Co., LTD Dr. Sinter Jr. for consolidation. The chamber was evacuated to 15 Pa and refilled with Ar (g) to 50,000 Pa to prevent H loss, and the sample was cold-pressed to 83 MPa. The pressure was reduced to 23 MPa before increasing the temperature to 900 °C in 25 min and then to 950 °C in 1 min, where it dwelled for 25 min. At 750 °C, the pressure was again increased to 83 MPa. The densities of the resulting pellets were determined via the Archimedes method to be >95% of the theoretical value.

### Powder X-ray Diffraction

After sintering, the pellets were polished with sandpaper and isopropanol to remove any surface oxide and transferred into a glovebox to be ground in an agate mortar and pestle and sieved for PXRD. The samples were removed from the glovebox and loaded onto a zero background X-ray plate via solvent smear (ethanol) for data collection in a Bruker D8 Eco Advance diffractometer with Cu Kα radiation operating at 40 kV and 25 mA. Diffraction data were collected from 2θ range 15–70° with a 0.015 step size and 1 s scan rate per step. Rietveld refinement employing the CIF^21^ for β-Yb_5_Sb_3_ was carried out using Topas5 software to refine lattice parameters and determine the phase purity of the samples. H position or occupancy was not refined. Therefore, the compositions, *x*, for Yb_5_Sb_3_H_*x*_ are nominal and taken from the reaction coefficients.

### Scanning Electron Microscopy (SEM) and Energy-Dispersive X-ray Spectroscopy (EDS)

The sintered pellets were cut with a Buehler Isomet diamond saw, and slices were mounted in epoxy pucks to be polished using sandpaper and a polishing wheel (1 μm colloidal diamond suspension) for SEM and EDS analysis. A Thermo Fisher Quattro ESEM instrument equipped with a Bruker Quantax EDX detector, an Everhart–Thornley detector, and an annular backscattered detector with 15 kV accelerating voltage was used to collect elemental maps and electron micrographs. The SEM micrographs and EDS elemental maps of Yb and Sb for the *x* = 0.25, 0.50, and 1.0 samples are provided in SI, Figure S3 and quantitative data in Table S4.

### Nuclear Magnetic Resonance

Solid-state ^1^H MAS NMR experiments were performed on a Bruker AVANCE 500 widebore NMR spectrometer equipped with an 11.74 T magnet and a 2.5 mm MAS probe. The powdered sample was loaded into a 2.5 mm Zirconia rotor with a Vespel cap. The rotor weight was measured before and after sample loading so that the mass of the powdered sample was directly associated with the ^1^H NMR signal intensity. The 2.5 mm rotor was span at 26 kHz. A direct polarization pulse sequence with a 30° tip angle and 5 s of recycle delay time was employed. A total of 128 scans were acquired for signal averaging, and 100 Hz line broadening was applied for data processing. The rotor background signal was also recorded at 26 kHz of MAS speed, and the background signal was deducted from the sample spectrum for data analysis. ^1^H MAS NMR spectra were obtained on YbH_2_, Yb_4_Sb_3_ prepared from arc-melted Yb metal and antimony, and samples of Yb_5_Sb_3_H_*x*_ prepared from Yb metal, antimony, and YbH_2_. The Yb_4_Sb_3_ sample did not provide a signal. The experimental ^1^H MAS NMR spectra of the 4 samples (*x* = 0.25–1.0) and an empty rotor are shown in SI, Figure S4. Details of the spectral deconvolution (SI, Table S5) and analysis are provided in the SI. The source of the background ^1^H signal could be from the probe stator and rotor cap. The ^1^H NMR signal intensities were normalized by the sample weight.

### Magnetization Measurements

Samples were inserted in a gel capsule held in a plastic straw and measured in a Quantum Design Magnetic Property Measurement System (QD-MPMS). Because of the presence of a ferromagnetic impurity in these samples similar to those of previous reports,^5,23^ the field dependence of the magnetization of the precursor YbH_2_ was also measured at room temperature. A small amount of soft ferromagnetic impurity was detected at 300 K in YbH_2_. The magnetic field-dependent data for YbH_2_ are shown in SI, Figure S5. If attributed to Fe, this would correspond to 0.004% Fe by mass in our YbH_2_ precursor. Therefore, to subtract the ferromagnetic impurity, we report the susceptibility of our samples as , similar to a previous study.^5^

### Thermoelectric Properties

Resistivity and Hall measurements were done using Van der Pauw geometry on a home-built system.^39^ Measurements were performed using a current of 0.1 A and magnetic field of 1 T. Ohmic contacts were checked using a voltage–current cure before measurement. Seebeck measurements were taken using a custom-built instrument^40^ in a low-pressure (300 Torr) N_2_ atmosphere from 300 to 775 K with multiple heating and cooling cycles. Graphite foil was placed between the sample and the thermocouple to ensure proper electrical contact. A Netzsch Laser Flash Analysis (LFA) Microflash 457 instrument was used for thermal diffusivity measurements and thermal conductivity was calculated with the equation the equation κ = λρ*C*_p_ where κ is the thermal conductivity, *C*_p_ is the Dulong–Petit heat capacity, λ is the thermal diffusivity, and ρ is the density of the sample. The experimental data are provided in the SI, Figures S6–S10, and sixth-order polynomial fits of the data are graphed in the main body of the manuscript.

## Results and Discussion

### Structure and Composition

The structure of orthorhombic Yb_5_Sb_3_H_*x*_ (Ca_5_Sb_3_F structure type (*Pnma*)) is shown in Figure 1. There are four Yb_4_ polyhedra in each unit cell that can accommodate either an interstitial H^–^ ion or an anionic electron.^7,8^ The polyhedra are separated by Sb^3–^ ions, as Sb–Sb distances are too long (∼4.5 Å) to be considered bonding interactions.^5^ Previous work has shown that all Yb cations are Yb^2+^, giving one excess electron per formula unit (5 × Yb^2+^ = 10 electrons donated, 3 × Sb^3–^ = 9 electrons accepted) and resulting in a formal valence state of (Yb_5_Sb_3_)^+^(*e*)^−^ unless H is incorporated, in which case the compound is charge-balanced as Yb_5_Sb_3_H.^5^

The orthorhombic phase is known to be stabilized by interstitial H. When synthesized with dehydrogenated metals, the hexagonal phase was produced, and when synthesized using YbH_2_, the orthorhombic phase was produced, and heating under vacuum at 1100 °C for 3 weeks caused a transition from the orthorhombic to the hexagonal phase.^7,8^ Because commercially available rare-earth metals are known to contain hydrogen impurities^38^ and experimental methods of quantifying H content in solid-state materials are limited, it is difficult to experimentally determine how much H is required to drive the structural transition from the hexagonal to the orthorhombic phase. Here, we employed total energy calculations to confirm that the orthorhombic phase is H-stabilized and to elucidate the minimum amount of H necessary to produce the orthorhombic structure.

**Figure 2:**
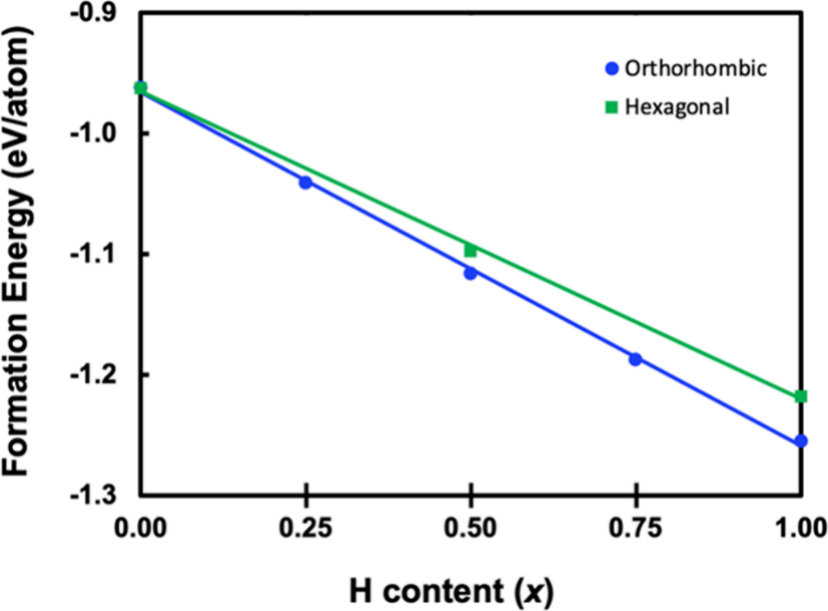
Formation energy, in eV/atom, for Yb_5_Sb_3_H_*x*_ in both the orthorhombic and hexagonal structure types as a function of the H content (*x*).

To investigate the chemical stability of the thermoelectric materials Yb_5_Sb_3_ and Yb_5_Sb_3_H_*x*_, the formation energies were calculated by density functional theory (DFT) and are shown in Figure 2. The formation energy is defined by eq 1.
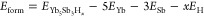
1

All formation energies are negative, which indicates that the compounds are chemically stable. There is only a slight difference in the formation energy of Yb_5_Sb_3_ in either the orthorhombic Ca_5_Sb_3_F (*Pnma*) or hexagonal Mn_5_Si_3_ (*P6*_*3*_*/mmc*) structure type by ∼10 meV/atom, with the hexagonal structure being more stable. Filling the interstitial sites of both structure types with H, following the stoichiometry of Yb_5_Sb_3_H_*x*_, significantly stabilizes the compounds. The more stable structure type switches from hexagonal to orthorhombic when any amount of H is incorporated. The presence of H likely stabilizes the orthorhombic structure type more so than the hexagonal structure, due to the tetrahedral nature of the interstitial site. The interstitial sites of the hexagonal structure type are octahedral, and thus the large volume is less favorable to H occupancy. This is consistent with other reports in which A_5_Sb_3_Z (A = Ca, Sr, Ba, Sm, Eu, Yb) compounds were found to crystallize in the orthorhombic *Pnma* space group for Z = H, F, and the hexagonal *P6*_*3*_*/mmc* for Z = Cl, Br.^6^ The orthorhombic and hexagonal structure types are competitive in their formation, which is due to the small difference in formation energies; however, it is possible to selectively form the orthorhombic phase by introducing interstitial H into the structure.

### Sample Characterization

**Figure 3:**
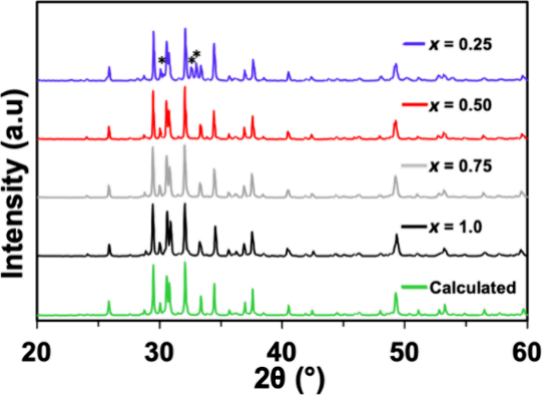
PXRD patterns of SPS’ed Yb_5_Sb_3_H_*x*_ (*x* = 0.25, 0.50, 0.75, 1.0) samples compared to the calculated pattern (bottom). The asterisks in *x* = 0.25 indicate peaks corresponding to the hexagonal Yb_5_Sb_3_H_*x*_ structure.

PXRD patterns for post-SPS’ed polycrystalline samples are shown in Figure 3, compared to the calculated pattern (bottom). The samples with higher H content (*x* = 0.50, 0.75, 1.0) can be refined as 100% orthorhombic structure type, and the lowest H-content sample (*x* = 0.25) contains ∼15% of the hexagonal phase. While the calculations above show that any amount of H will stabilize the orthorhombic phase, samples were synthesized in sealed Ta tubes which are permeable to H_2_ at high temperatures,^41,42^ making it likely that not all H was incorporated into the compound, resulting in the hexagonal impurity. Previous experimental work on orthorhombic Ca_5_Sb_3_H_*x*_ supports this hypothesis, as 0.5 equiv of H was necessary in a welded Ta container to produce >95% yield of the orthorhombic structure type.^28^ This is consistent with other studies on A_5_Pn_3_ systems that have shown intermediate H content leads to the hexagonal and orthorhombic phases existing in equilibrium.^6,27−30^ Samples with higher H content (*x* = 0.50, 0.75, 1.0) are 100% orthorhombic Yb_5_Sb_3_H_*x*_.

**Figure 4:**
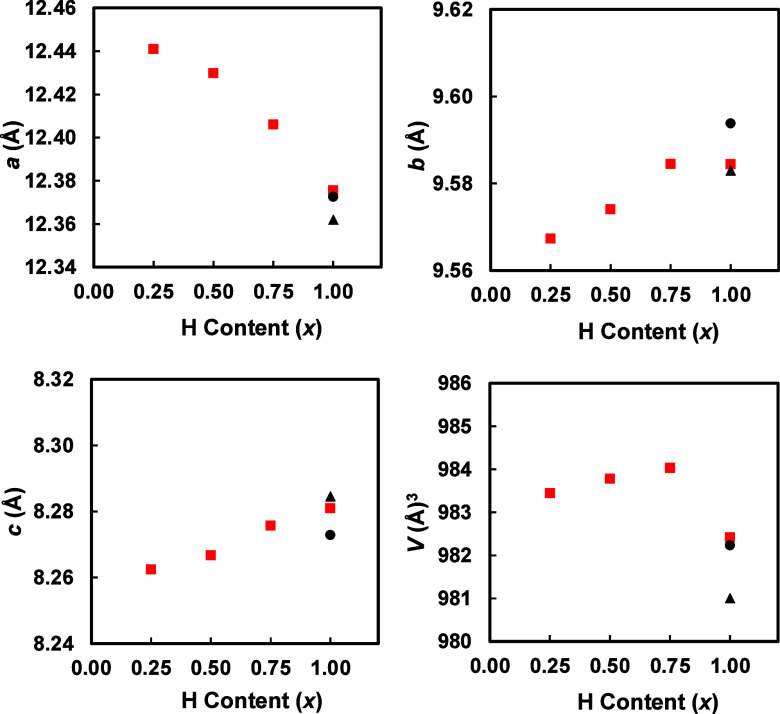
Lattice parameters and volume (red square) versus nominal *x* of Yb_5_Sb_3_H_*x*_ (*x* = 0.25, 0.50, 0.75, 1.0) determined via Rietveld refinement of PXRD data as a function of H content compared with results from ref (28) (black square) and ref (5) (black triangle).

Lattice parameters determined via Rietveld refinement in red compared with the published parameters in black^28,5^ for Yb_5_Sb_3_H_*x*_ are given in Figure 4 (statistics in SI, Figure S1 and Table S2). The *a* lattice parameter decreases sharply with an increasing H content, while the *c* parameter increases slightly. The *b* parameter increases slightly from *x* = 0.25–0.75 and remains the same for *x* = 1.0. This results in the volume increasing from *x* = 0.25–0.75 with a decrease in volume for *x* = 1.0. The black symbols show the published lattice parameters^28^ and while *a* and volume are consistent, the *b* and *c* show an opposite trend. It was proposed that with full H, the structure contracts, observed in all filled hydride phases of this type.^28^ Previously calculated electron densities showed oblong isosurfaces in the interstitial sites that extend in the crystallographic *a* direction.^5^ The decreasing *a* parameter is consistent with a decreased level of electronic repulsion that would accompany a shift from a confined electron in the interstitial site to a H atom. The systematic change in lattice parameters, in particular, the *a* lattice parameter, is an indication that H content varies between samples, although our *x* values are nominal, taken from the reaction coefficients. However, this result supports previous findings of a decreased cell volume with H content^28,5^ in orthorhombic Yb_5_Sb_3_H and the unexpectedly small lattice constants in other Mn_5_Si_3_-type phases due to interstitial H impurities.^6,7^ Other studies on the A_5_Pn_3_ (A = Ca, Sr, Ba, Eu, Yb; Pn = Sb, Bi) compounds in the Ca_5_Sb_3_F structure type have used unit cell volume to estimate the amount of H in each sample.^6^

**Figure 5:**
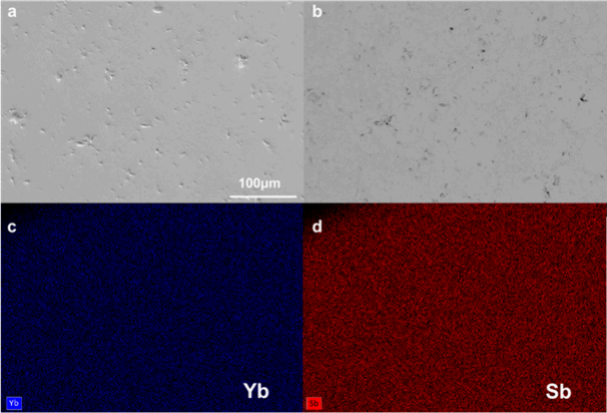
(a) Secondary electron SEM micrograph, (b) backscattered electron SEM micrograph, (c) Yb EDS elemental map, and (d) Sb EDS elemental map for Yb_5_Sb_3_H_*x*_, *x* = 0.75. The scale bar of 100 μm shown in panel (a) is the same for all panels.

Figure 5 depicts typical SEM micrographs and EDS elemental maps of Yb and Sb for the *x* = 0.75 sample and in SI, Figure S3, for the remaining samples. Backscattered electron images and elemental analysis confirm phase purity and uniform distribution of elements.

**Figure 6:**
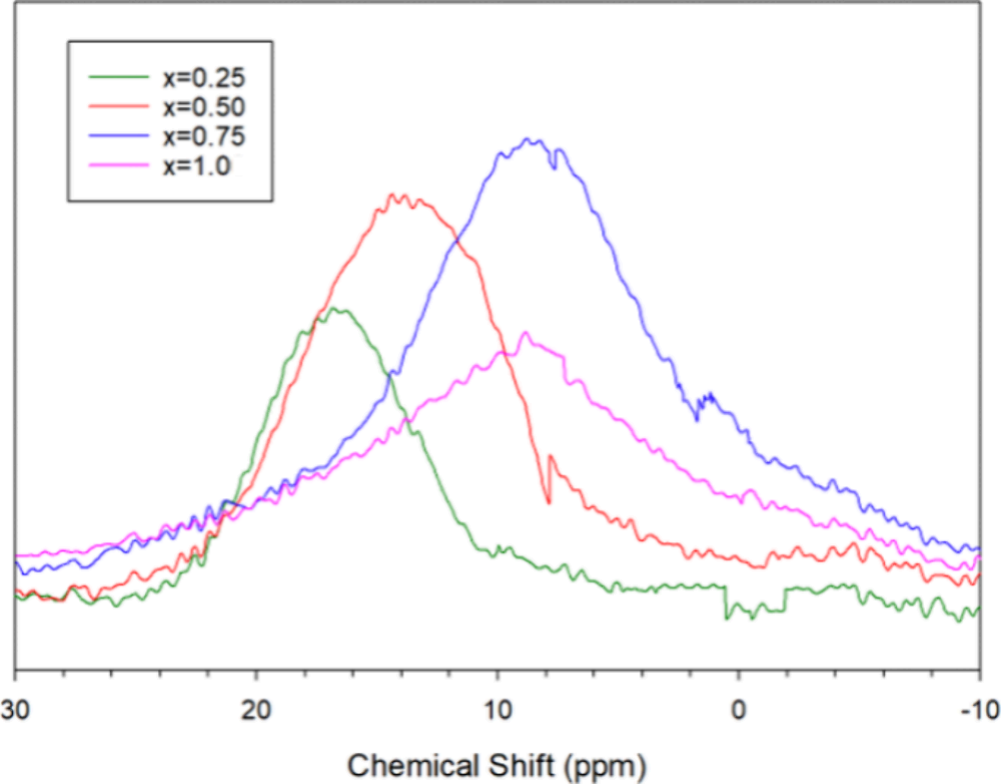
^1^H NMR spectra of Yb_5_Sb_3_H_*x*_ (*x* = 0.25, 0.50, 0.75, 1.0) in the isotropic chemical shift region with instrument background deduction and sample weight normalization.

**Figure 7:**
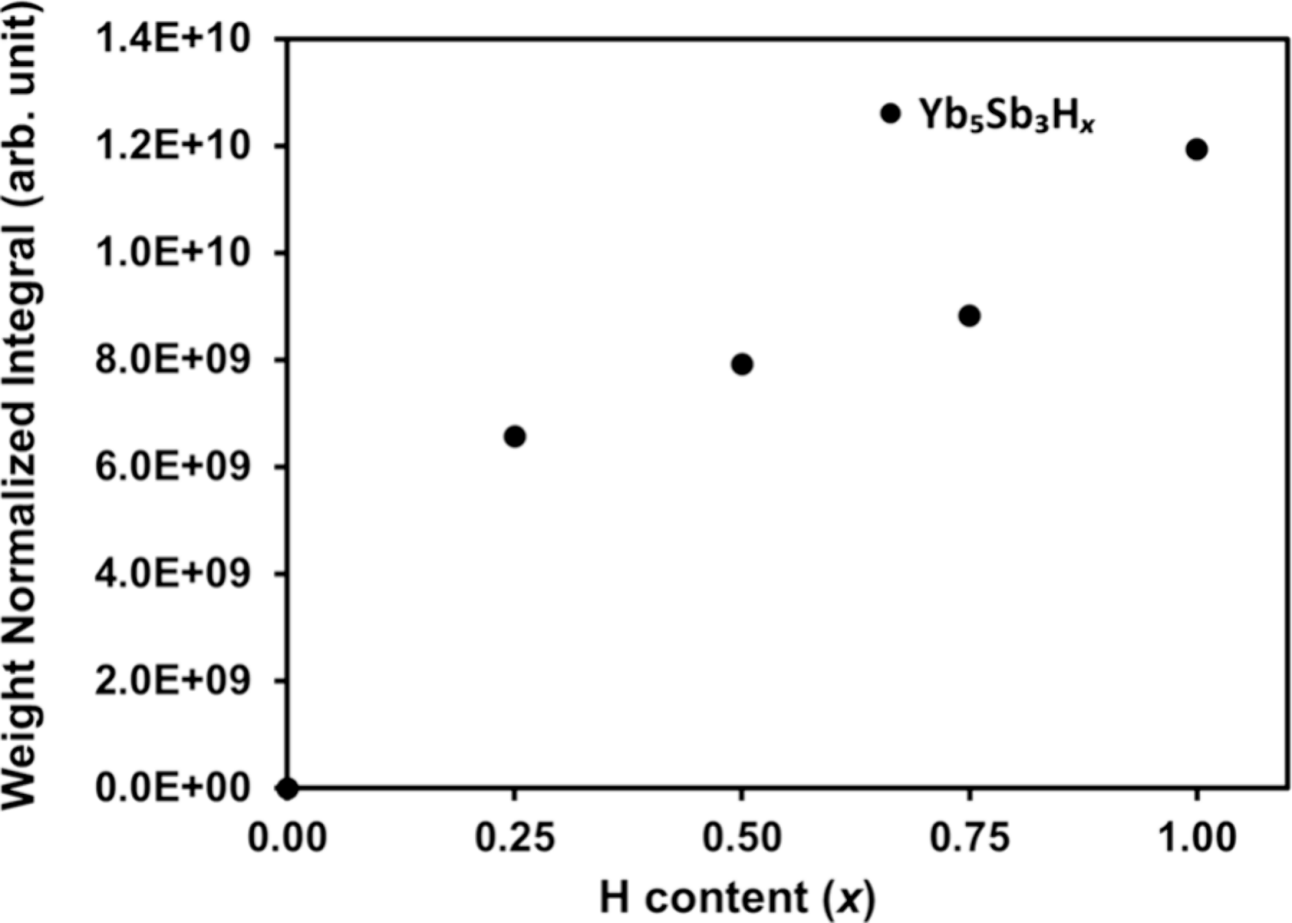
^1^H MAS NMR signal intensity vs hydrogen content of Yb_5_Sb_3_H_*x*_ (*x* = 0.25, 0.50, 0.75, 1.0).

After subtraction of the instrument background, the ^1^H MAS NMR spectra from the samples are displayed in Figure 6. ^1^H NMR chemical shifts of metal hydrides are often in the range of 0–60 ppm,^43,44^ and PbH_2_ is known to have a ^1^H NMR shift of 31.3 ppm.^45^^1^H NMR signal intensities for these samples have been normalized by the sample weight. The isotropic chemical shift of the ^1^H NMR signals shifts to higher fields with increasing *x* values in the Yb_5_Sb_3_H_*x*_ samples, from 17 ppm in Yb_5_Sb_3_H_0.25_ to 9.7 ppm in Yb_5_Sb_3_H_0.75_ and Yb_5_Sb_3_H_1_. This shift suggests shielding of the H that would be expected from a shorter metal hydride bond, consistent with the shorter Yb–H distances indicated by the decreasing *a* lattice parameter with increasing H content. With increasing H content, the *a* lattice parameter becomes smaller and the Yb–H bonds become shorter and stronger. Changing bond strengths may impact defect chemistry, as has been observed in other Zintl phases.^46^ The peak width also increases with increasing *x* value. Broader lineshapes are caused by deviations in local magnetic fields that can be attributed to structural disorder and imperfect crystalline packing.^47^ The ^1^H MAS NMR signal integrations (after background subtraction and sample weight normalization) are listed in Table 1 and are shown as a function of *x* in Figure 7.

**Table 1:** ^1^H MAS NMR Results

sample	integral (arb. unit)^a^	chemical shift (ppm)	full width at half height (Hz)
Yb_5_Sb_3_H_0.25_	6.567 × 10^09^ (∼ 5%)^b^	16.6	4198
Yb_5_Sb_3_H_0.50_	7.922 × 10^09^ (∼ 5%)	14.2	5292
Yb_5_Sb_3_H_0.75_	8.822 × 10^09^ (∼ 5%)	10.0	6542
Yb_5_Sb_3_H_1.0_	1.193 × 10^10^ (∼ 5%)	9.7	8846

a Integral is the ^1^H NMR signal intensity of the Yb_5_Sb_3_H_*x*_ spectrum deducting the instrument background, then normalized with regard to the sample weight. Integral = (experimental data – fitted background)/weight.

b Integral uncertainty, the uncertainty with the experimental spectrum is based on the overlapping parameter for simulation, which is 95%. The uncertainty with the sample weight is determined by the balance, which is 0.0001 g. In this study, the weight uncertainty is 0.0001/0.0600 = 0.00167. The final uncertainty is [(spectrum_uncertainty)^2^ + (weight_uncertainty)^2^]^0.5^.

### Magnetic Measurements

**Figure 8:**
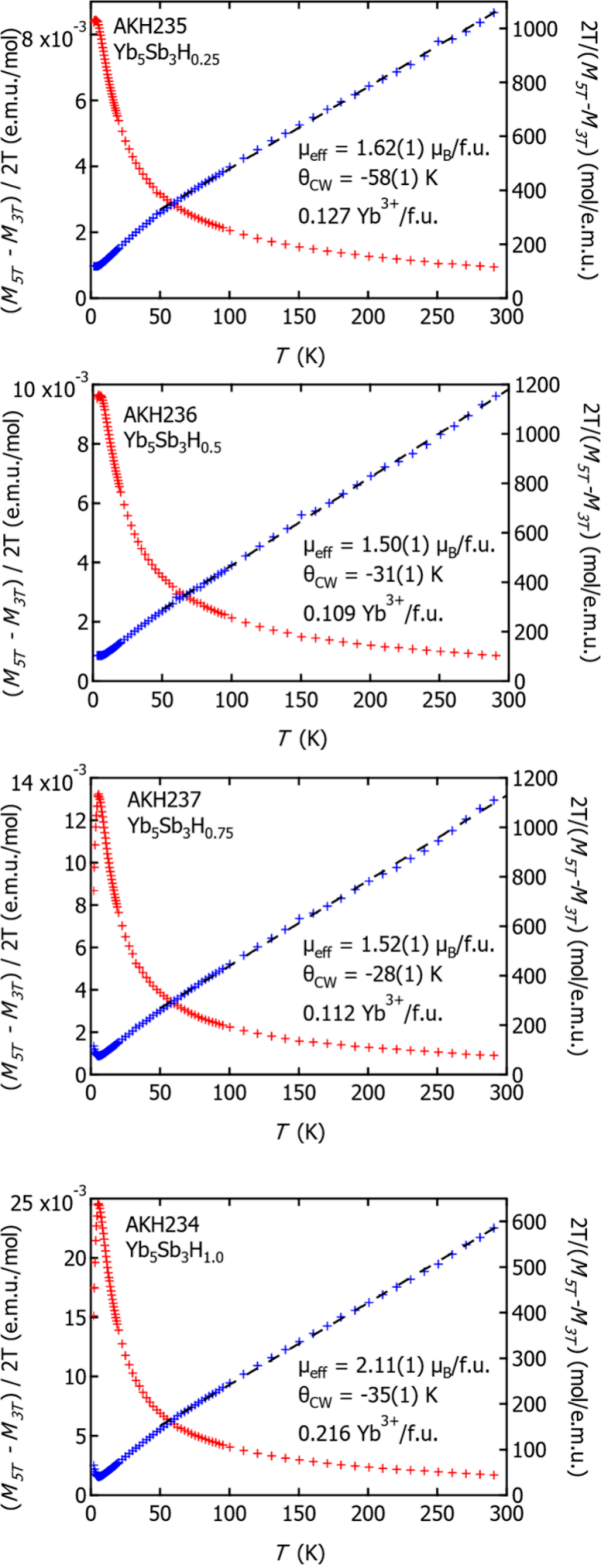
Magnetic susceptibility (red) and inverse susceptibility (blue) plots with fits from 50 to 300 K (black) of Yb_5_Sb_3_H_*x*_ (*x* = 0.25, 0.50, 0.75, 1.0).

The susceptibility measurements are listed in Figure 8. Upon cooling, the susceptibility increases (the saturation or peak observed at 5 K is an artifact due to the ferromagnetic impurity subtraction). We fit the inverse susceptibility with a Curie–Weiss law to extract the effective moments and Curie–Weiss temperature. The Weiss constant is −53 K for *x* = 0.25 and is reduced to ∼ −30 K for *x* > 0.25, with the local moment changing from 1.6 μ_B_ to ∼2.0 μ_B_ with increasing *x*. Electronic structure calculations suggest that the anionic electron provides a localized magnetic moment, and Yb_5_Sb_3_ can be described as a Mott-insulating electride. In the case of the orthorhombic (β) phase, Yb_5_Sb_3_(*e*^*–*^) vs Yb_5_Sb_3_H, the electrons are localized at the interstitial sites, and there is polarization of the Yb_4_ tetrahedra that could induce orbital hybridization. Our results are similar to those reported by Lu et al.^5^ where they also observe a negative Weiss constant suggesting antiferromagnetic interactions of the spin with a small overall moment. They explain the Curie–Weiss temperature dependence and localized moment, supported by additional theoretical calculations,^4^ as Coulomb correlations between the localized anionic electrons which give rise to the observed electronic and magnetic properties of β-Yb_5_Sb_3_H_*x*_. However, another possible hypothesis is that as the bonding changes with the inclusion of a hydride the defect energies of the sites are affected. We obtain the broadest line width in ^1^H NMR with *x* = 1.0, suggesting structural disorder or defects might be present. If we attribute the effective moments to a small amount of Yb^3+^ with a theoretical effective moment of 4.54 *μ*_*B*_, then we find that 0.1–0.2 Yb^3+^/Yb_5_Sb_3_H_*x*_ is enough to explain the observed Curie–Weiss behavior in all samples. However, adding more electrons with Yb^3+^ is consistent with the observed carrier concentration trends (described below) only if there is a defect that compensates. It is also possible that the Yb^3+^ is from a surface oxide, as we noticed that the samples tarnish over time in air. Further investigations of the hydride-containing compositions with a pair distribution function and Yb NMR would provide additional insight.

### Thermoelectric Property Measurements

**Figure 9:**
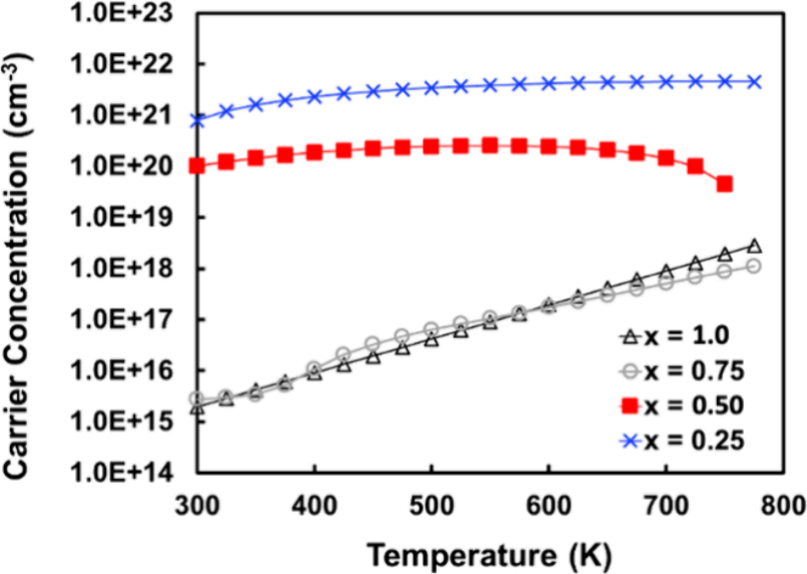
Charge carrier concentration determined via the Hall effect from 300 to 775 K for Yb_5_Sb_3_H_*x*_ (*x* = 0.25, 0.50, 0.75, and 1.0).

**Figure 10:**
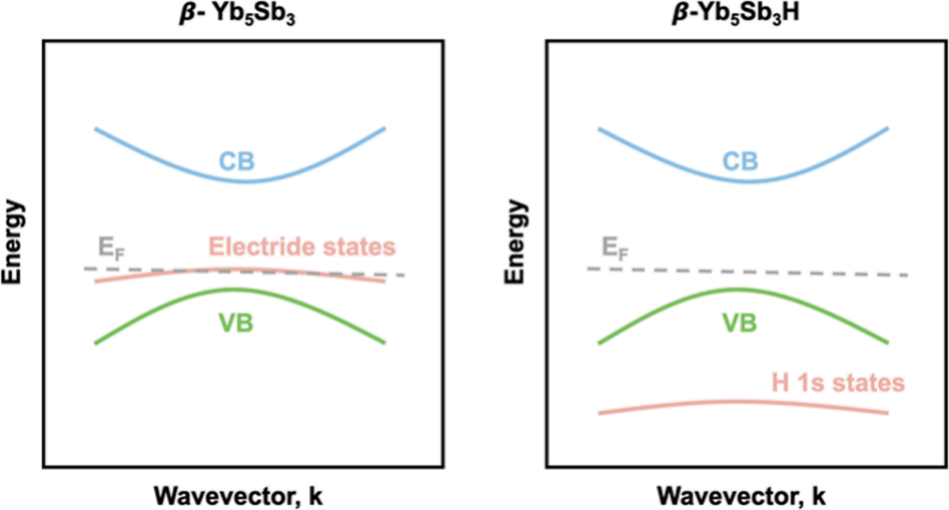
Schematic of the band structures^5^ reported for Yb_5_Sb_3_H_*x*_ (*x* = 0, 1) showing the position of the electride and hydride states.

Hall measurements show that all samples are *p*-type and the carrier concentration decreases by several orders of magnitude as H content is increased (Figure 9), from ∼8 × 10^20^ cm^–3^ for *x* = 0.25 to ∼2 × 10^15^ cm^–3^ for *x* = 1.0 at 300 K, indicating that H incorporation is an effective way to tune electronic properties. The gap between samples shown in Figure 9 suggests that there should be a critical concentration of H content where the electride states still exist at a modest concentration (i.e., 50%) but are not observable in the Hall measurements due to their extreme localization (i.e., a metal–insulator transition). Band structure calculations^5^ on Yb_5_Sb_3_H_*x*_ show the presence of four localized electride bands at the Fermi level for *x* = 0, that are replaced by four localized H 1s states ∼4 eV below the Fermi level for Yb_5_Sb_3_H_*x*_ when *x* = 1.0 (the number of electride bands changes with *x* so that there are as many electride bands as unoccupied interstitial sites). See the schematic of the published band structure^5^ in Figure 10. Except for the states attributed to the electride/hydride, the other valence and conduction bands do not change significantly as *x* varies. The Fermi level for both Yb_5_Sb_3_ and Yb_5_Sb_3_H is near the valence band;^5^ hence, it is not surprising that we see p-type behavior in our measurements. Therein, for Yb_5_Sb_3_, the Fermi level crosses quite deeply through the valence band and *E*_g_ is smaller, consistent with the high carrier concentration observed. For Yb_5_Sb_3_H, the Fermi level is located on the edge of the valence band (almost in the band gap), with quite large *E*_g_, which explains the more insulating behavior we observe in the experiment for samples with large H content. Rather than conceptualizing the excess electrons as *n*-type dopants added to the conduction band, they are retained as anions in localized bands in 0D cavities with similar dispersion as the H 1s states. With this scenario, the decrease in carrier concentration as H content increases is consistent with changes in band structure. However, it is also possible that the dominant source of free holes in the system is Yb^2+^ vacancies, as is common in other ytterbium antimonides,^46,48,49^ the presence of H inside the tetrahedral Yb_4_ cages is likely to impact equilibrium defect concentration. Calculations show (Figure 2) that the orthorhombic structure becomes more stable as H content increases, and the compound becomes charge-balanced at *x* = 1.0. In the absence of H interstitials, Yb^2+^ vacancies could serve as a charge-compensating mechanism to reduce the number of anionic electrons and stabilize the structure. ^1^H MAS NMR data shows H peaks shifting upfield as H content increases and the PXRD shows that the *a* lattice constant becomes smaller with increasing H. Both phenomena are consistent with shorter, stronger Yb–H bonding, which could lead to greater defect formation energy for Yb vacancies and thereby lower carrier concentration as *x* increases.

**Figure 11:**
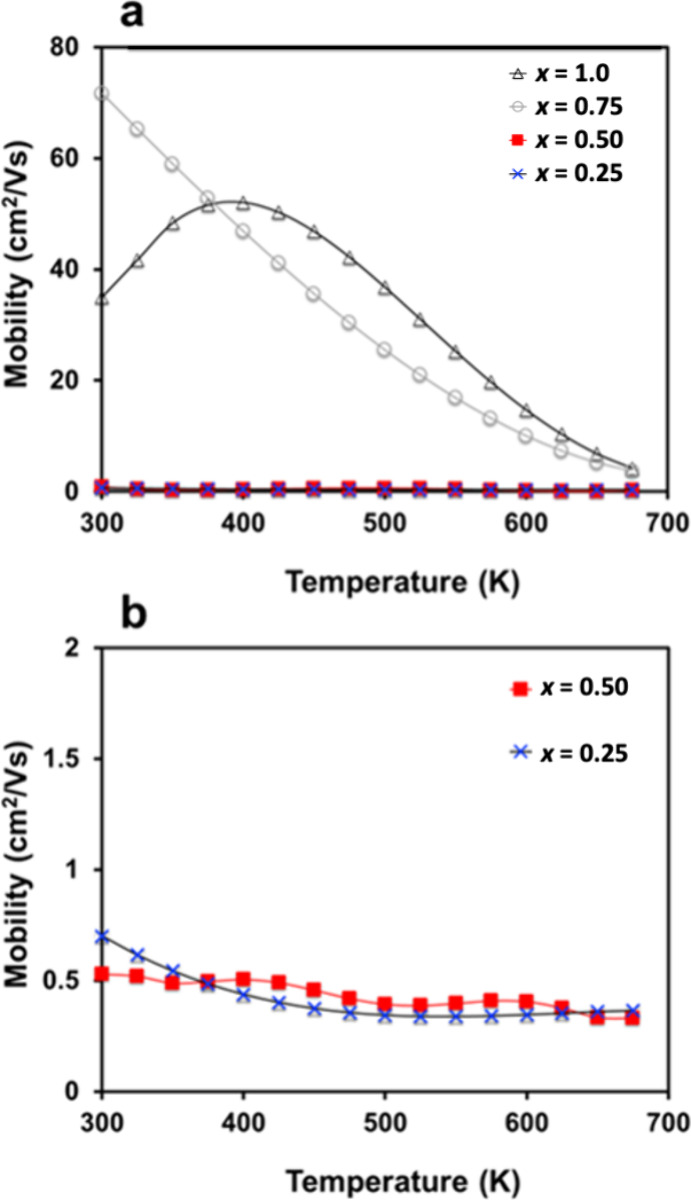
(a) Charge carrier mobility from 300 to 775 K for Yb_5_Sb_3_H_*x*_, for Yb_5_Sb_3_H*_x_, x* = 0.25, 0.50, 0.75, 1.0 and (b) plot showing the temperature dependence of charge carrier mobility for Yb_5_Sb_3_H_*x*,_*x* = 0.25, 0.50.

The charge carrier mobility (Figure 11) increases dramatically as H is incorporated, that is, as the electride bands are removed, from ∼0.5 cm^2^/(V s) to ∼70 cm^2^/(V s) at 300 K. For *x* = 1.0, thermally activated mobility is observed below 400 K, which is often an indication of grain boundary scattering that reduces mobility at low temperatures. As discussed above, when electride bands are present, as expected for samples with *x* < 1, the trapped electrons are confined in 0D interstitial cavities that have similar symmetry to H 1s orbitals just below the Fermi level, resulting in low charge carrier mobility. High mobility has been reported in other, higher dimensional electrides, but the 0D nature of the tetrahedral holes in this system results in low mobility. The charge carrier mobility increases dramatically as the localized electride bands are removed (*i.e. x* approaches 1) and the Fermi level moves into the valence band, which is primarily composed of Sb 5*p* states, with some contribution from Yb 5*d* orbitals, according to calculated DOS.^5^

**Figure 12:**
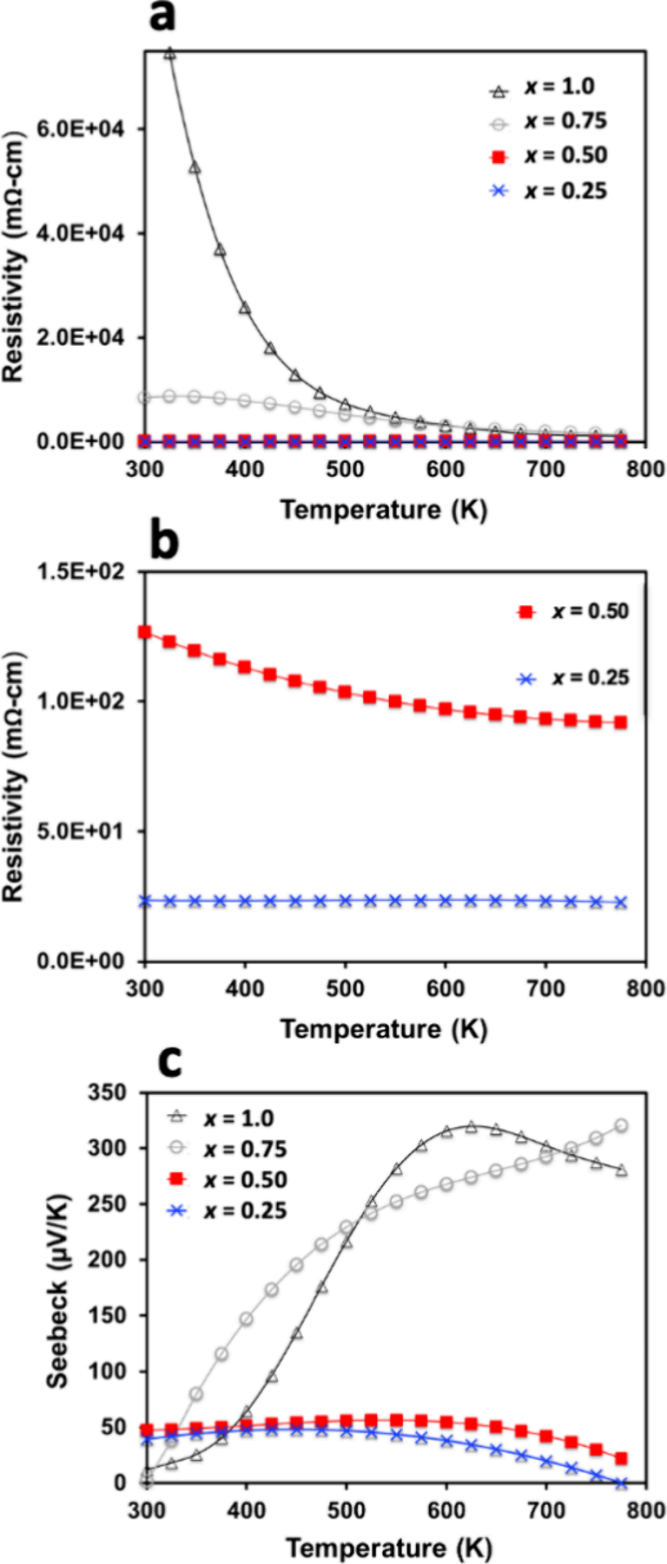
Temperature-dependent (a, b) electrical resistivity and (c) Seebeck coefficient from 300 to 775 K for Yb_5_Sb_3_H_*x*_ (*x* = 0.25, 0.50, 0.75, 1.0).

Electrical resistivity (Figure 12a,b) trends as expected, decreasing as the carrier concentration increases. The room temperature resistivity for *x* = 1.0, ∼74,000 mΩ cm at 325 K, is insulating as expected from the low carrier concentration (∼10^15^ cm^–3^) and consistent with high values previously reported,^5^ while a more moderate value of 23.4 mΩ cm is observed for *x* = 0.25 due to the high carrier concentration and extremely low mobility. Seebeck coefficient (Figure 12c) also trends as expected based on carrier concentration data, with *x* = 1 exhibiting the highest value of 330 μV/K at 650 K and then decreasing to 775 K, most likely due to the increasing carrier concentration with temperature. The lowest Seebeck coefficient is observed for *x* = 0.25, which reaches a maximum value of 48.2 μV/K at 450 K before decreasing due to an increasing carrier concentration. The lower onset temperature of bipolar conduction for *x* = 0.25 compared to *x* = 1.0 could be explained by the smaller band gap in *x* = 0.25 due to the presence of electride bands ∼0.5 eV above the valence band maximum. In previous work, experimentally determined band gaps of Yb_5_Sb_3_F were smaller than calculated values, suggesting that a small amount of anionic electron states survived due to F deficiencies, which could explain the decreasing Seebeck coefficient for *x* = 0.75 and 1 at relatively low temperatures given that the gap calculated via DFT is 0.8 eV.^5^

The electronic properties fall into two distinct groups, with *x* = 0.25 and 0.50 giving high carrier concentration and low mobility, resistivity, and Seebeck coefficient with similar temperature dependence, behaving more like 0D electrides, while *x* = 0.75 and 1.0 exhibit low carrier concentration and high mobility, resistivity and Seebeck coefficient, behaving more like lightly doped charge-balanced semiconductors. This trend is supported by calculations that show the number of electride bands corresponds to the number of vacant interstitial sites.^5^

**Figure 13:**
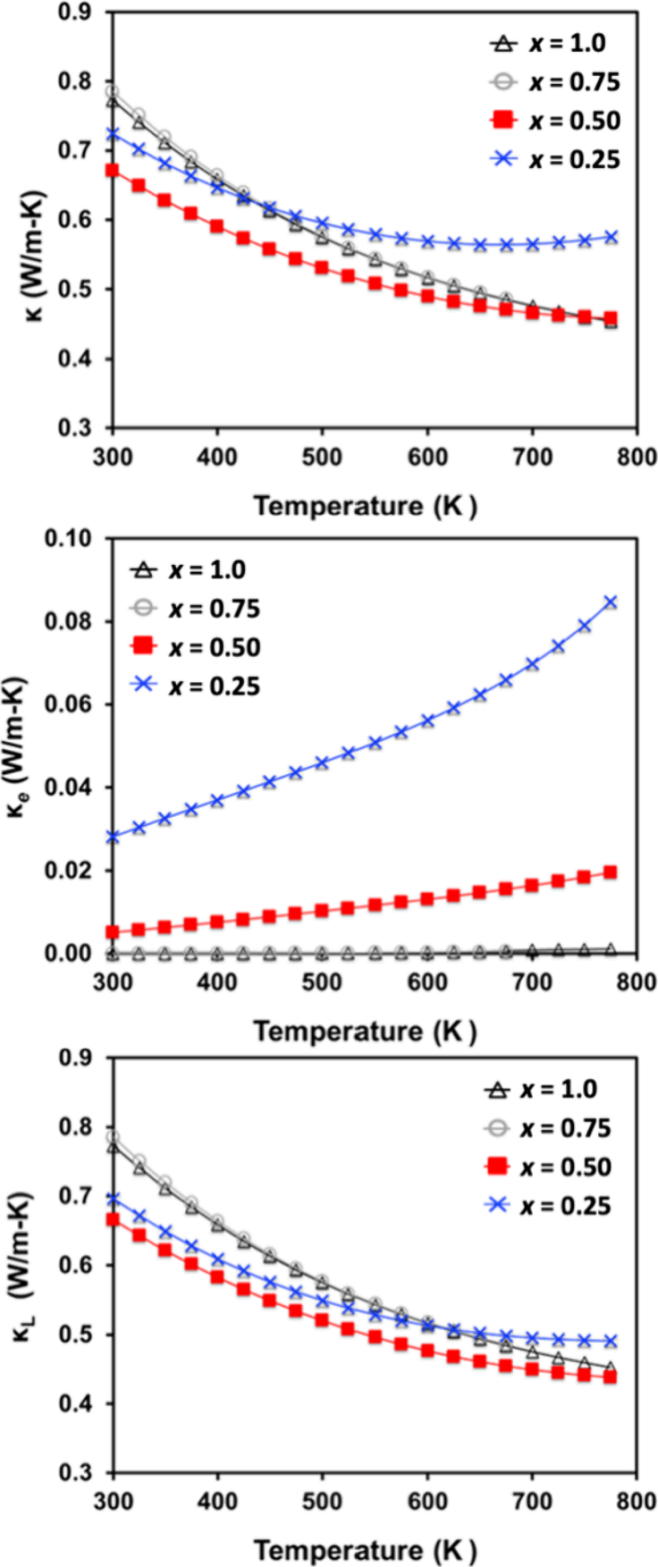
Total thermal conductivity (top); electronic component of thermal conductivity (middle) and lattice thermal conductivity (bottom) for Yb_5_Sb_3_H_*x*_ (*x* = 0.25, 0.50, 0.75, 1.0).

Thermal conductivity is shown in Figure 13 and was calculated from experimentally determined thermal diffusivity and heat capacity (Dulong–Petit law). Values are ultralow for all samples, < 0.8 W/mK from 300 to 775 K, as expected for a compound containing heavy atoms and six unique crystallographic sites. The electronic contribution of the thermal conductivity was calculated using the Lorenz number derived from the Seebeck coefficient^50^ and was negligible for *x* = 0.75 and 1.0 due to the high electrical resistivity. Lattice thermal conductivity is lower for samples with a lower H content, which could be due to phonon scattering at the electride site. The lowest lattice thermal is observed from *x* = 0.50, which has the greatest configurational entropy given its half-filled H site.

**Figure 14:**
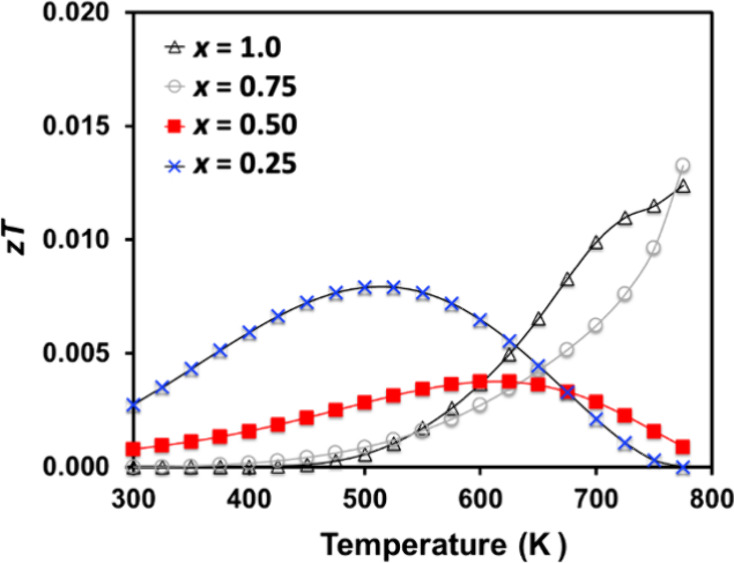
Thermoelectric figure of merit as a function of temperature for Yb_5_Sb_3_H_*x*_ (*x* = 0.25, 0.50, 0.75, and 1.0).

The thermoelectric figure of merit, *zT*, was low for all samples (Figure 14), reaching a maximum value of ∼0.013 at 725 K for *x* = 0.75 and 1.0. Given the high charge carrier mobility and low lattice thermal conductivity of *x* = 1.0, a higher *zT* may be achieved if the carrier concentration is increased. Significant value peak *zT* is usually reached at carrier concentrations between 10^19^ and 10^21^ cm^–3^, similar in value for the low H content electrides but several orders of magnitude higher than the high H content semiconductors we report here. Band structure calculations reveal three bands just below the Fermi level in *x* = 1.0, two heavy bands, and one light. The combination of heavy and light bands and high valley degeneracy is favorable for thermoelectric performance and it is likely that better thermoelectric efficiency can be designed through chemical substitutions.^51−53^

## Conclusions

We have reported the thermoelectric properties for Yb_5_Sb_3_H_*x*_ (*x* = 0.25, 0.50, 0.75, 1.0) and characterized the structural and electronic changes as the H content increases, causing a transition from an electride semimetal to a charge-balanced semiconductor. The samples exhibit extremely low charge carrier mobility at low H content (x = 0.25, 0.50), consistent with anionic electrons localized in 0D cavities. Magnetization shows Curie–Weiss behavior for all samples with a small moment and antiferromagnetic coupling that could be attributed to the electride or a small amount of Yb^3+^ that arises from either a change in the defect energy or surface oxidation. Compositions with low H content (*x* = 0.25 and 0.50) have low mobility and moderate electrical resistivity due to the high carrier concentration that may arise from defects in the structure. The mobility increases dramatically with H content (*x* = 0.75, 1.0) as the localized electride bands near the Fermi level are replaced with lower-lying bands with similar dispersion to H 1s orbitals, causing a transition to semiconducting behavior with charge transport dominated by delocalized states.^5^ The highest *zT* was observed for *x* = 1.0 due to high charge carrier mobility, high Seebeck coefficient, and low lattice thermal conductivity. Given the localized 0D nature of the electride cavities in orthorhombic Yb_5_Sb_3_H, the mobility is too low for applications in thermoelectrics, but higher *zT* is likely to be achievable for the semiconducting *x* = 1.0 material due to its excellent mobility if the carrier concentration is able to be increased, for example, via *p-*type doping with an alkali metal. This work highlights the importance of systematically probing structure–property relationships and shows that minor changes in defects and composition can dramatically impact structure stability and electronic properties. It opens the door to the use of interstitial impurities, and especially H, to tune electronic properties in thermoelectrics, as well as other functional materials.

## Supporting Information Available

Final atomic positions for the relaxed structures, Rietveld refinements of Yb_5_Sb_3_H_*x*_ PXRD data, statistics for Rietveld refinements of Yb_5_Sb_3_H_*x*_ PXRD data, Rietveld refinements of Yb_4_Sb_3_ PXRD data, statistics for Rietveld refinements of Yb_4_Sb_3_ PXRD data, SEM micrographs and EDS elemental maps and data, ^1^H MAS NMR spectra of Yb_5_Sb_3_H_*x*_, magnetization data for the YbH_2_ precursor, experimental electrical resistivity data, Seebeck data, carrier concentration and mobility data, and thermal conductivity data (PDF)
