# Supplementary material for: β-Phase Yb5Sb3Hx: Magnetic and Thermoelectric Properties Traversing from an Electride to a Semiconductor
Source: Inorg Chem. 2024 Apr 23;63(18):8109–19. doi: 10.1021/acs.inorgchem.4c00254 (PMC11080061; doi:10.1021/acs.inorgchem.4c00254)
Supplement: Supplementary file 1 — ic4c00254_si_001.pdf [file ic4c00254_si_001.pdf]

# Supporting Information

## *β*-phase $Yb_5Sb_3H_x$: Magnetic and Thermoelectric Properties Traversing from Electride to Semiconductor

**Ashlee K. Hauble,[1] Tanner Q. Kimberly,[1] Kamil M. Ciesielski,[2] Nicholas Mrachek,[1] Maxwell G. Wright,[3] Valentin Taufour,[3] Ping Yu,[4] Eric S. Toberer,[2] and Susan M. Kauzlarich[1*]**

**Affiliations**

[1]Department of Chemistry, University of California, One Shields Ave, Davis, CA 95616, United States[2]Department of Physics, Colorado School of Mines, 1500 Illinois St, Golden, CO 80401, United States

[3]Department of Physics and Astronomy, University of California, One Shields Ave, Davis, CA 95616, United States

[4]Nuclear Magnetic Resonance Facility, University of California, One Shields Ave, Davis, CA 95616, United States

*Corresponding author's email: smkauzlarich@ucdavis.edu

**Table of Contents**

**Table S1**. Atomic Coordinates of $Yb_5Sb_3H_x$ after Structure Relaxation Calculations

**Figure S1**. Rietveld refinements of $Yb_5Sb_3H_x$ PXRD data

**Table S2**. Statistics for Rietveld refinements of $Yb_5Sb_3H_x$ PXRD data

**Figure S2**. Rietveld refinements of $Yb_4Sb_3$ PXRD data

**Table S3**. Statistics for Rietveld refinements of $Yb_4Sb_3$ PXRD data

**Figure S3**. SEM micrographs and EDS elemental maps

**Table S4**. EDS data

**Figure S4**. $^1H$ MAS NMR spectra of $Yb_5Sb_3H_x$

**Table S5**. $^1H$ MAS NMR spectral fitting parameters

**Figure S5**. Magnetic susceptibility and analysis of $YbH_2$ powder

**Figure S6**. Experimental electrical resistivity data

**Figure S7**. Experimental Seebeck data

**Figure S8**. Experimental carrier concentration data

**Figure S9**. Experimental mobility data

**Figure S10**. Experimental thermal conductivity data

**Table S1**. Atomic Coordinates of $Yb_5Sb_3H_x$ after Structure Relaxation Calculations

**$Yb_5Sb_3$ hexagonal**

ATOMIC_POSITIONS (Crystal Coordinates)

| | | | |
|---|---|---|---|
| Yb2 | 0.3333333430 | 0.6666666870 | 0.0000000000 |
| Yb2 | 0.6666666270 | 0.3333333130 | -0.0000000000 |
| Yb1 | 0.6666666270 | 0.3333333130 | 0.5000000000 |
| Yb1 | 0.3333333430 | 0.6666666870 | 0.5000000000 |
| Yb | 0.2529560691 | 0.0000000000 | 0.2499992103 |
| Yb | 0.7470439009 | 0.0000000000 | 0.7500007897 |
| Yb | 0.0000000000 | 0.2529560691 | 0.2499992103 |
| Yb | -0.0000000000 | 0.7470439009 | 0.7500007897 |
| Yb | 0.7470439009 | 0.7470439009 | 0.2499992103 |
| Yb | 0.2529560691 | 0.2529560691 | 0.7500007897 |
| Sb | 0.6097462829 | -0.0000000000 | 0.2499998896 |
| Sb | 0.3902537171 | 0.0000000000 | 0.7500001104 |
| Sb | -0.0000000000 | 0.6097462829 | 0.2499998896 |
| Sb | 0.0000000000 | 0.3902537171 | 0.7500001104 |
| Sb | 0.3902537171 | 0.3902537171 | 0.2499998896 |
| Sb | 0.6097462829 | 0.6097462829 | 0.7500001104 |

**$Yb_5Sb_3H_{0.5}$ hexagonal**

ATOMIC_POSITIONS (Crystal Coordinates)

| | | | |
|---|---|---|---|
| Yb2 | 0.3333333430 | 0.6666666870 | 0.0000000000 |
| Yb2 | 0.6666666270 | 0.3333333130 | -0.0000000000 |
| Yb1 | 0.6666666270 | 0.3333333130 | 0.5000000000 |
| Yb1 | 0.3333333430 | 0.6666666870 | 0.5000000000 |
| Yb | 0.2463145238 | -0.0000000000 | 0.2405195761 |

| | | | |
|---|---|---|---|
| Yb | 0.7536854462 | 0.0000000000 | 0.7594804239 |
| Yb | 0.0000000000 | 0.2463145238 | 0.2405195761 |
| Yb | -0.0000000000 | 0.7536854462 | 0.7594804239 |
| Yb | 0.7536854462 | 0.7536854462 | 0.2405195761 |
| Yb | 0.2463145238 | 0.2463145238 | 0.7594804239 |
| Sb | 0.6087863188 | -0.0000000000 | 0.2499139496 |
| Sb | 0.3912136812 | 0.0000000000 | 0.7500860504 |
| Sb | 0.0000000000 | 0.6087863188 | 0.2499139496 |
| Sb | -0.0000000000 | 0.3912136812 | 0.7500860504 |
| Sb | 0.3912136812 | 0.3912136812 | 0.2499139496 |
| Sb | 0.6087863188 | 0.6087863188 | 0.7500860504 |
| H | -0.0000000000 | -0.0000000000 | -0.0000000000 |

**$Yb_5Sb_3H$ hexagonal**

ATOMIC_POSITIONS (Crystal Coordinates)

| | | | |
|---|---|---|---|
| Yb2 | 0.3333333430 | 0.6666666870 | 0.0000000000 |
| Yb2 | 0.6666666270 | 0.3333333130 | 0.0000000000 |
| Yb1 | 0.6666666270 | 0.3333333130 | 0.5000000000 |
| Yb1 | 0.3333333430 | 0.6666666870 | 0.5000000000 |
| Yb | 0.2422341314 | 0.0000000000 | 0.2499999719 |
| Yb | 0.7577658386 | -0.0000000000 | 0.7500000281 |
| Yb | -0.0000000000 | 0.2422341314 | 0.2499999719 |
| Yb | 0.0000000000 | 0.7577658386 | 0.7500000281 |
| Yb | 0.7577658386 | 0.7577658386 | 0.2499999719 |
| Yb | 0.2422341314 | 0.2422341314 | 0.7500000281 |
| Sb | 0.6081774984 | 0.0000000000 | 0.2500000058 |
| Sb | 0.3918225016 | -0.0000000000 | 0.7499999942 |
| Sb | 0.0000000000 | 0.6081774984 | 0.2500000058 |
| Sb | -0.0000000000 | 0.3918225016 | 0.7499999942 |
| Sb | 0.3918225016 | 0.3918225016 | 0.2500000058 |
| Sb | 0.6081774984 | 0.6081774984 | 0.7499999942 |

| | | | |
|---|---|---|---|
| H | -0.0000000000 | -0.0000000000 | 0.5000000000 |
| H | -0.0000000000 | -0.0000000000 | 0.0000000000 |

**$Yb_5Sb_3$ orthorhombic**

ATOMIC_POSITIONS (Crystal Coordinates)

| | | | |
|---|---|---|---|
| Yb1 | 0.0752540001 | 0.0417456356 | 0.1952098235 |
| Yb2 | 0.9247459765 | 0.9582544370 | 0.8047904146 |
| Yb2 | 0.4247459765 | 0.9582544370 | 0.6952095854 |
| Yb1 | 0.5752540001 | 0.0417456356 | 0.3047901765 |
| Yb2 | 0.9247459765 | 0.5417455630 | 0.8047904146 |
| Yb1 | 0.0752540001 | 0.4582543534 | 0.1952098235 |
| Yb1 | 0.5752540001 | 0.4582543534 | 0.3047901765 |
| Yb2 | 0.4247459765 | 0.5417455630 | 0.6952095854 |
| Yb | 0.2288762325 | 0.2500000000 | 0.8182345943 |
| Yb | 0.7711236759 | 0.7500000000 | 0.1817655825 |
| Yb | 0.2711236759 | 0.7500000000 | 0.3182344175 |
| Yb | 0.7288762325 | 0.2500000000 | 0.6817654057 |
| Yb1 | 0.2908689520 | 0.2500000000 | 0.3457806005 |
| Yb2 | 0.7091311164 | 0.7500000000 | 0.6542192407 |
| Yb2 | 0.2091311454 | 0.7500000000 | 0.8457807593 |
| Yb1 | 0.7908689810 | 0.2500000000 | 0.1542193995 |
| Yb1 | 0.0044154543 | 0.2500000000 | 0.5364727798 |
| Yb2 | 0.9955843536 | 0.7500000000 | 0.4635275504 |
| Yb2 | 0.4955843536 | 0.7500000000 | 0.0364724496 |
| Yb1 | 0.5044154563 | 0.2500000000 | 0.9635272202 |
| Sb | 0.3285968350 | 0.4878696997 | 0.0636540647 |
| Sb | 0.6714032444 | 0.5121308078 | 0.9363459514 |
| Sb | 0.1714032444 | 0.5121308078 | 0.5636540486 |
| Sb | 0.8285968350 | 0.4878696997 | 0.4363459433 |
| Sb | 0.6714032444 | 0.9878691922 | 0.9363459514 |
| Sb | 0.3285968350 | 0.0121303003 | 0.0636540647 |

| | | | |
|---|---|---|---|
| Sb | 0.8285968350 | 0.0121303003 | 0.4363459433 |
| Sb | 0.1714032444 | 0.9878691922 | 0.5636540486 |
| Sb | 0.4837064748 | 0.2500000000 | 0.5775446368 |
| Sb | 0.5162935975 | 0.7500000000 | 0.4224553251 |
| Sb | 0.0162935975 | 0.7500000000 | 0.0775446749 |
| Sb | 0.9837064748 | 0.2500000000 | 0.9224553632 |

**$Yb_5Sb_3H_{0.25}$ orthorhombic**

ATOMIC_POSITIONS (Crystal Coordinates)

| | | | |
|---|---|---|---|
| Yb1 | 0.0737375108 | 0.0417248338 | 0.1955785468 |
| Yb2 | 0.9247439659 | 0.9574683278 | 0.8034116230 |
| Yb2 | 0.4258391506 | 0.9577307175 | 0.6958239673 |
| Yb1 | 0.5747342957 | 0.0421302639 | 0.3039298440 |
| Yb2 | 0.9247439659 | 0.5425316722 | 0.8034116230 |
| Yb1 | 0.0737375108 | 0.4582751552 | 0.1955785468 |
| Yb1 | 0.5747342957 | 0.4578697251 | 0.3039298440 |
| Yb2 | 0.4258391506 | 0.5422692825 | 0.6958239673 |
| Yb | 0.2290632753 | 0.2500000000 | 0.8181649671 |
| Yb | 0.7700482886 | 0.7500000000 | 0.1821969928 |
| Yb | 0.2703029727 | 0.7500000000 | 0.3188343170 |
| Yb | 0.7294000895 | 0.2500000000 | 0.6811612573 |
| Yb1 | 0.2901269333 | 0.2500000000 | 0.3455632684 |
| Yb2 | 0.7135731414 | 0.7500000000 | 0.6514334452 |
| Yb2 | 0.2110362111 | 0.7500000000 | 0.8480698750 |
| Yb1 | 0.7902080808 | 0.2500000000 | 0.1518116823 |
| Yb1 | 0.0042301517 | 0.2500000000 | 0.5362824378 |
| Yb2 | 0.9924267533 | 0.7500000000 | 0.4656792331 |
| Yb2 | 0.4957048351 | 0.7500000000 | 0.0362133271 |
| Yb1 | 0.5044074559 | 0.2500000000 | 0.9632931394 |
| Sb | 0.3285169275 | 0.4878309755 | 0.0646373769 |
| Sb | 0.6713721001 | 0.5128391567 | 0.9339590373 |

| | | | |
|---|---|---|---|
| Sb | 0.1702996661 | 0.5134139672 | 0.5648794742 |
| Sb | 0.8282772432 | 0.4854944179 | 0.4319830382 |
| Sb | 0.6713721001 | 0.9871608433 | 0.9339590373 |
| Sb | 0.3285169275 | 0.0121690245 | 0.0646373769 |
| Sb | 0.8282772432 | 0.0145055821 | 0.4319830382 |
| Sb | 0.1702996661 | 0.9865860328 | 0.5648794742 |
| Sb | 0.4829275718 | 0.2500000000 | 0.5778707306 |
| Sb | 0.5172734458 | 0.7500000000 | 0.4219778154 |
| Sb | 0.0160134192 | 0.7500000000 | 0.0780195822 |
| Sb | 0.9837468190 | 0.2500000000 | 0.9218499987 |
| H | 0.8940688127 | 0.7500000000 | 0.6944721158 |

$Yb_5Sb_3H_{0.50}$ orthorhombic

ATOMIC_POSITIONS (Crystal Coordinates)

| | | | |
|---|---|---|---|
| Yb1 | 0.0742972201 | 0.0426682159 | 0.1962010445 |
| Yb2 | 0.9257026620 | 0.9573317209 | 0.8037988060 |
| Yb2 | 0.4256646719 | 0.9576160969 | 0.6960373734 |
| Yb1 | 0.5743353543 | 0.0423838234 | 0.3039628019 |
| Yb2 | 0.9257026620 | 0.5426682791 | 0.8037988060 |
| Yb1 | 0.0742972201 | 0.4573317731 | 0.1962010445 |
| Yb1 | 0.5743353543 | 0.4576161656 | 0.3039628019 |
| Yb2 | 0.4256646719 | 0.5423839031 | 0.6960373734 |
| Yb | 0.2295928879 | 0.2500000000 | 0.8177512860 |
| Yb | 0.7704071898 | 0.7500000000 | 0.1822484602 |
| Yb | 0.2700009936 | 0.7500000000 | 0.3193208231 |
| Yb | 0.7299990573 | 0.2500000000 | 0.6806793129 |
| Yb1 | 0.2860852631 | 0.2500000000 | 0.3483826457 |
| Yb2 | 0.7139147764 | 0.7500000000 | 0.6516175043 |
| Yb2 | 0.2112412826 | 0.7500000000 | 0.8503048912 |
| Yb1 | 0.7887587034 | 0.2500000000 | 0.1496948859 |
| Yb1 | 0.0074058232 | 0.2500000000 | 0.5342233004 |

| | | | |
|---|---|---|---|
| Yb2 | 0.9925939867 | 0.7500000000 | 0.4657768543 |
| Yb2 | 0.4956894729 | 0.7500000000 | 0.0365947842 |
| Yb1 | 0.5043107294 | 0.2500000000 | 0.9634050598 |
| Sb | 0.3286977406 | 0.4870010178 | 0.0663528426 |
| Sb | 0.6713024502 | 0.5129989775 | 0.9336469802 |
| Sb | 0.1705842141 | 0.5158023184 | 0.5687194827 |
| Sb | 0.8294155905 | 0.4841976905 | 0.4312806807 |
| Sb | 0.6713024502 | 0.9870010225 | 0.9336469802 |
| Sb | 0.3286977406 | 0.0129989822 | 0.0663528426 |
| Sb | 0.8294155905 | 0.0158023095 | 0.4312806807 |
| Sb | 0.1705842141 | 0.9841976816 | 0.5687194827 |
| Sb | 0.4822923102 | 0.2500000000 | 0.5778746692 |
| Sb | 0.5177077908 | 0.7500000000 | 0.4221255446 |
| Sb | 0.0160927852 | 0.7500000000 | 0.0787218859 |
| Sb | 0.9839071531 | 0.2500000000 | 0.9212778874 |
| H | 0.8941382724 | 0.7500000000 | 0.6947466299 |
| H | 0.1058617128 | 0.2500000000 | 0.3052535669 |

**$Yb_5Sb_3H_{0.75}$ orthorhombic**

ATOMIC_POSITIONS (Crystal Coordinates)

| | | | |
|---|---|---|---|
| Yb1 | 0.0738881335 | 0.0430312208 | 0.1963874860 |
| Yb2 | 0.9254190843 | 0.9571101758 | 0.8035154938 |
| Yb2 | 0.4265641234 | 0.9576088685 | 0.6956622763 |
| Yb1 | 0.5750667808 | 0.0428743779 | 0.3029447984 |
| Yb2 | 0.9254190843 | 0.5428898242 | 0.8035154938 |
| Yb1 | 0.0738881335 | 0.4569687682 | 0.1963874860 |
| Yb1 | 0.5750667808 | 0.4571256111 | 0.3029447984 |
| Yb2 | 0.4265641234 | 0.5423911315 | 0.6956622763 |
| Yb | 0.2299602198 | 0.2500000000 | 0.8182027767 |
| Yb | 0.7703228398 | 0.7500000000 | 0.1816067630 |
| Yb | 0.2703127102 | 0.7500000000 | 0.3192089271 |

| | | | |
|---|---|---|---|
| Yb | 0.7305251953 | 0.2500000000 | 0.6812992252 |
| Yb1 | 0.2851237650 | 0.2500000000 | 0.3506275810 |
| Yb2 | 0.7136077868 | 0.7500000000 | 0.6501940313 |
| Yb2 | 0.2118060963 | 0.7500000000 | 0.8499567933 |
| Yb1 | 0.7855874037 | 0.2500000000 | 0.1481241146 |
| Yb1 | 0.0070988847 | 0.2500000000 | 0.5346192220 |
| Yb2 | 0.9924443829 | 0.7500000000 | 0.4650183541 |
| Yb2 | 0.4957647056 | 0.7500000000 | 0.0365115624 |
| Yb1 | 0.5075194110 | 0.2500000000 | 0.9649276908 |
| Sb | 0.3298036092 | 0.4855177662 | 0.0668463693 |
| Sb | 0.6715737343 | 0.5150830469 | 0.9302953388 |
| Sb | 0.1705751120 | 0.5158489421 | 0.5689109234 |
| Sb | 0.8294038861 | 0.4836607387 | 0.4299549470 |
| Sb | 0.6715737343 | 0.9849169531 | 0.9302953388 |
| Sb | 0.3298036092 | 0.0144822338 | 0.0668463693 |
| Sb | 0.8294038861 | 0.0163392613 | 0.4299549470 |
| Sb | 0.1705751120 | 0.9841510579 | 0.5689109234 |
| Sb | 0.4829434286 | 0.2500000000 | 0.5779250174 |
| Sb | 0.5175434573 | 0.7500000000 | 0.4213738841 |
| Sb | 0.0167970917 | 0.7500000000 | 0.0785565740 |
| Sb | 0.9832786024 | 0.2500000000 | 0.9211595970 |
| H | 0.8938111830 | 0.7500000000 | 0.6934150244 |
| H | 0.1056346211 | 0.2500000000 | 0.3061891117 |
| H | 0.6057293067 | 0.2500000000 | 0.1933484839 |

**$Yb_5Sb_3H$ orthorhombic**

ATOMIC_POSITIONS (Crystal Coordinates)

| | | | |
|---|---|---|---|
| Yb1 | 0.0740005281 | 0.0430661773 | 0.1964624756 |
| Yb2 | 0.9259995230 | 0.9569337846 | 0.8035373154 |
| Yb2 | 0.4259995230 | 0.9569337846 | 0.6964626846 |
| Yb1 | 0.5740005281 | 0.0430661773 | 0.3035375244 |

| | | | |
|---|---|---|---|
| Yb2 | 0.9259995230 | 0.5430662154 | 0.8035373154 |
| Yb1 | 0.0740005281 | 0.4569338117 | 0.1964624756 |
| Yb1 | 0.5740005281 | 0.4569338117 | 0.3035375244 |
| Yb2 | 0.4259995230 | 0.5430662154 | 0.6964626846 |
| Yb | 0.2301938638 | 0.2500000000 | 0.8188231509 |
| Yb | 0.7698062456 | 0.7500000000 | 0.1811767431 |
| Yb | 0.2698062456 | 0.7500000000 | 0.3188232569 |
| Yb | 0.7301938638 | 0.2500000000 | 0.6811768491 |
| Yb1 | 0.2850974889 | 0.2500000000 | 0.3517133655 |
| Yb2 | 0.7149024347 | 0.7500000000 | 0.6482867922 |
| Yb2 | 0.2149024637 | 0.7500000000 | 0.8517132078 |
| Yb1 | 0.7850975179 | 0.2500000000 | 0.1482866345 |
| Yb1 | 0.0072212167 | 0.2500000000 | 0.5348859855 |
| Yb2 | 0.9927787501 | 0.7500000000 | 0.4651140328 |
| Yb2 | 0.4927787501 | 0.7500000000 | 0.0348859672 |
| Yb1 | 0.5072212187 | 0.2500000000 | 0.9651140145 |
| Sb | 0.3294375248 | 0.4836263826 | 0.0701158171 |
| Sb | 0.6705624748 | 0.5163736973 | 0.9298841553 |
| Sb | 0.1705624748 | 0.5163736973 | 0.5701158447 |
| Sb | 0.8294375248 | 0.4836263826 | 0.4298841909 |
| Sb | 0.6705624748 | 0.9836263027 | 0.9298841553 |
| Sb | 0.3294375248 | 0.0163736174 | 0.0701158171 |
| Sb | 0.8294375248 | 0.0163736174 | 0.4298841909 |
| Sb | 0.1705624748 | 0.9836263027 | 0.5701158447 |
| Sb | 0.4827693968 | 0.2500000000 | 0.5787777371 |
| Sb | 0.5172305696 | 0.7500000000 | 0.4212224853 |
| Sb | 0.0172305696 | 0.7500000000 | 0.0787775147 |
| Sb | 0.9827693968 | 0.2500000000 | 0.9212222629 |
| H | 0.8944483804 | 0.7500000000 | 0.6935329465 |
| H | 0.1055515209 | 0.2500000000 | 0.3064669582 |
| H | 0.6055515209 | 0.2500000000 | 0.1935330418 |

| | | | |
|---|---|---|---|
| H | 0.3944483804 | 0.7500000000 | 0.8064670535 |

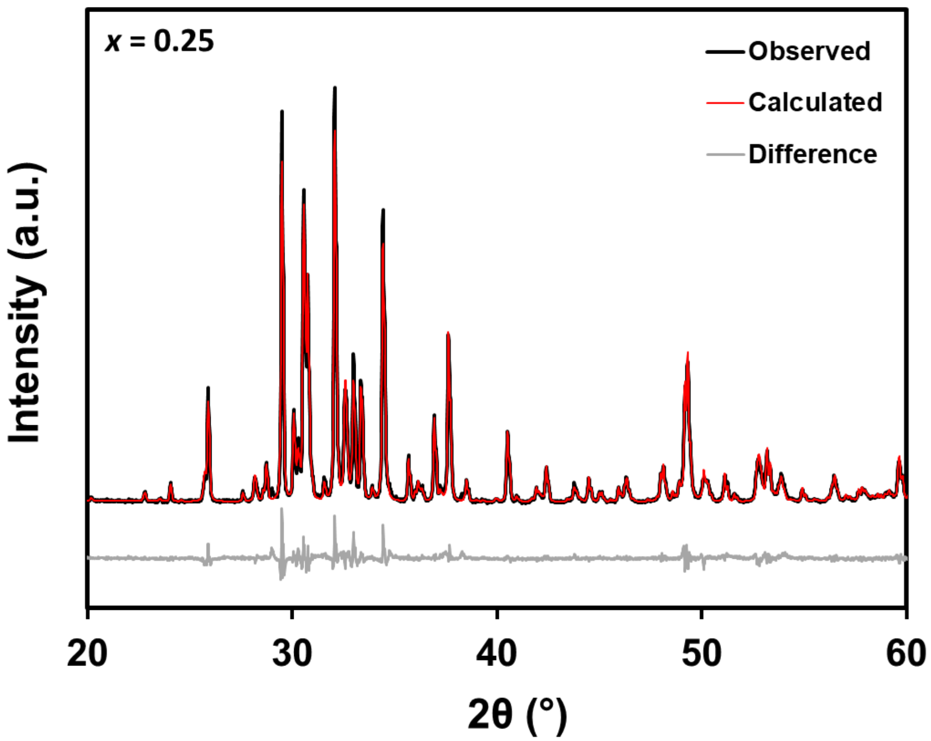

x = 0.25
Observed
Calculated
Difference
Intensity (a.u.)
20
30
40
50
60
2θ (°)


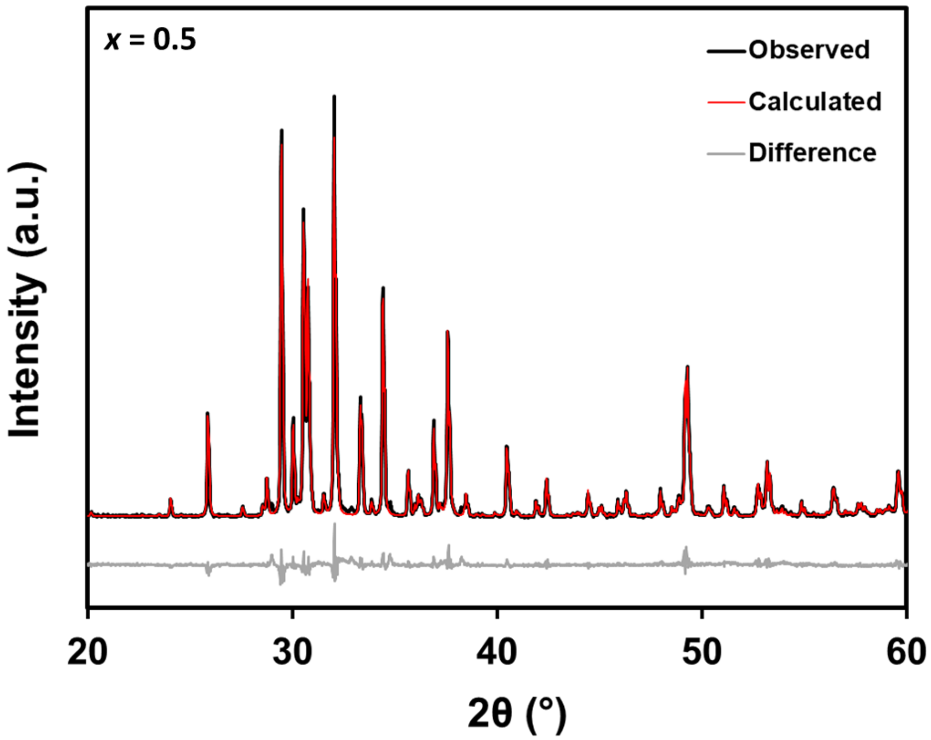

x = 0.5
Observed
Calculated
Difference
Intensity (a.u.)
20
30
40
50
60
2θ (°)

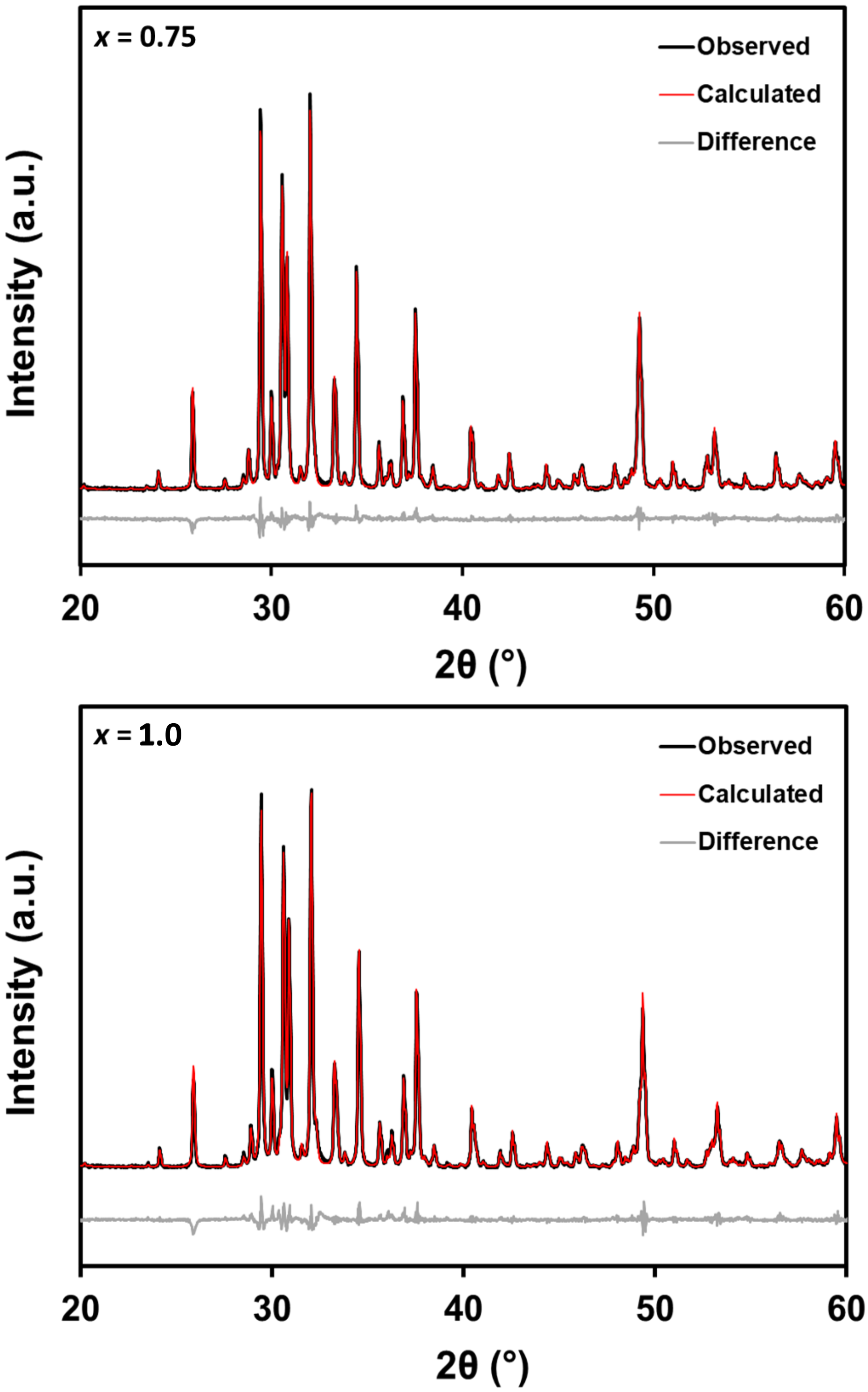


**Figure S1**. Portion of typical Rietveld refinements of $Yb_5Sb_3H_x$ ($x$ = 0.25, 0.50, 0.75, 1.0). The observed data are shown in black, calculated pattern in red, and difference curve is in gray.

**Table S2**. Selected Rietveld Refinement Statistics for $Yb_5Sb_3H_x$[a]

| *x* | | **0.25** | **0.50** | **0.75** | **1.0** |
|---|---|---|---|---|---|
| **Unit Cell Parameters** | *a* (Å) | 12.4409(2) | 12.4298(2) | 12.4061(2) | 12.3725(2) |
| | *b* (Å) | 9.5673(2) | 9.5741(2) | 9.5845(2) | 9.5844(2) |
| | *c* (Å) | 8.2624(2) | 8.2668(2) | 8.2757(1) | 8.2846(2) |
| | *V* (Å$^3$) | 983.45(3) | 983.78(3) | 984.04(3) | 982.42(3) |
| **Rp (%), Rwp (%)** | | 7.10, 9.48 | 7.42, 10.48 | 5.65, 7.71 | 6.07, 8.22 |
| **Orthorhombic type (wt %)** | | 79.15 | 100 | 100 | 100 |
| **Hexagonal type (wt %)** | | 20.85 | 0 | 0 | 0 |
| **From ref 28** | *a* (Å) | | | | 12.3756(9) |
| | *b* (Å) | | | | 9.5938(4) |
| | *c* (Å) | | | | 8.2729(4) |
| | *V* (Å$^3$) | | | | 982.24(4) |
| **From ref 5** | *a* (Å) | | | | 12.362(2) |
| | *b* (Å) | | | | 9.583(5) |
| | *c* (Å) | | | | 8.281(1) |
| | *V* (Å$^3$)$^+$ | | | | 981.01(3) |

[a]Data from 15-70 ˚ 2θ

$^+$Calculated from lattice parameters

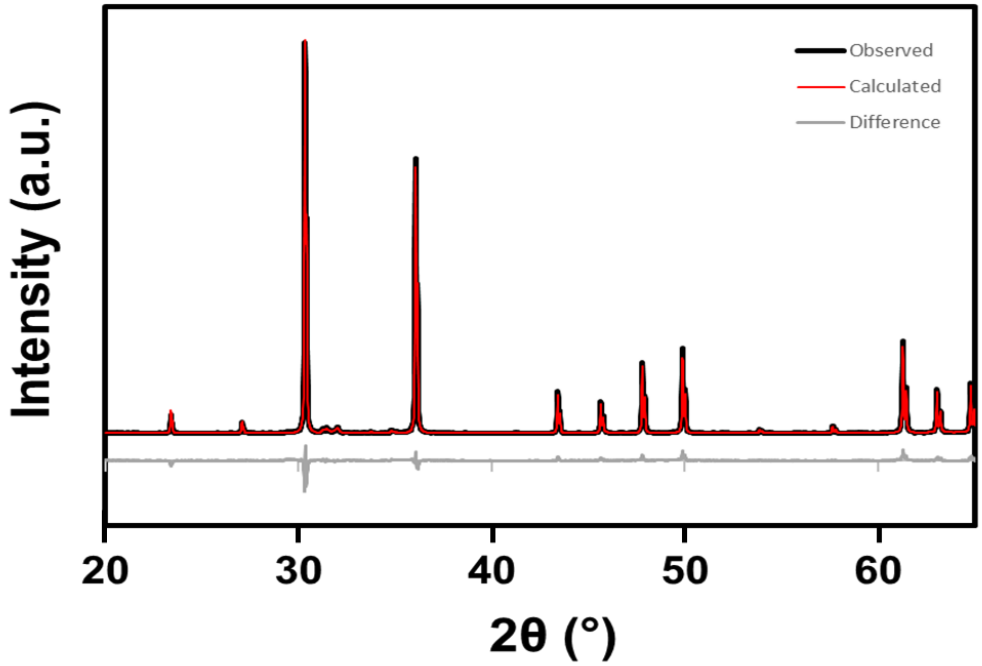


**Figure S2**. A portion of a typical Rietveld refinement of the $Yb_4Sb_3$ precursor.

**Table S3**. Selected Rietveld Refinement Statistics from $Yb_4Sb_3$ precursor[a]

| ***x*** | | |
|---|---|---|
| **Unit Cell Parameters** | *a* (Å) | 9.32397(1) |
| | *V* (Å$^3$) | 810.59(2) |
| **Rp (%), Rwp (%)** | 9.21 | 11.90 |
| **$Yb_4Sb_3$ (wt %)** | 93.90 | |
| **$Yb_{11}Sb_{10}$ (wt %)** | 6.10 | |

[a]Data collected from 15-70 ° 2θ

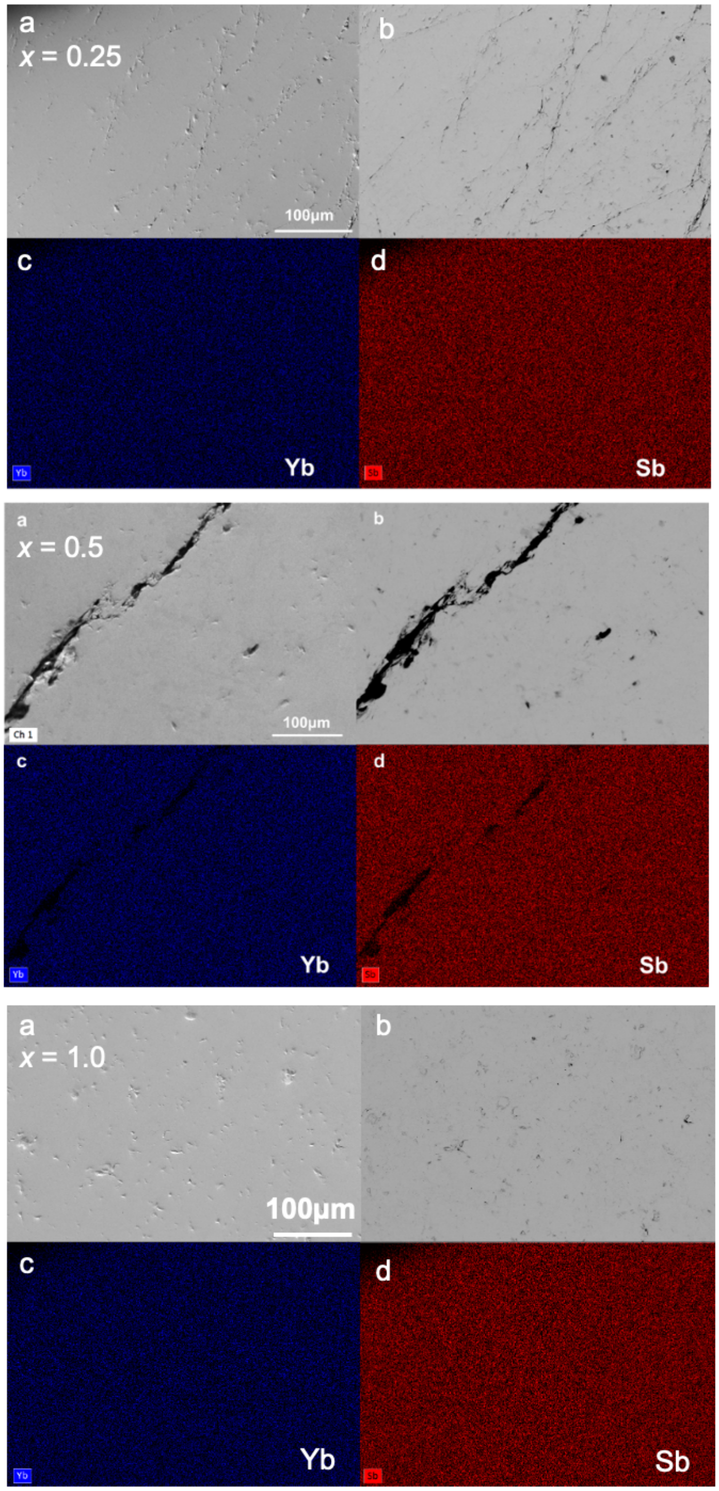


**Figure S3**. (a) Secondary electron SEM micrograph, (b) backscattered electron SEM micrograph, (c) Yb EDS elemental map, (d) Sb EDS elemental map for $Yb_5Sb_3H_x$, $x = 0.25$ (top), $x = 0.50$ (middle), and $x = 1.00$ (bottom). The scale bar shown in panel a is the same for all panels.

**Table S4.** EDS data for $Yb_5Sb_3H_x$ (H cannot be detected).

| | Yb at. % | Sb at. % |
|---|---|---|
| Calc.$Yb_5Sb_3$ | 62.5 | 37.5 |
| $Yb_5Sb_3H_{0.25}$ | 62.7(9) | 37.3(9) |
| $Yb_5Sb_3H_{0.5}$ | 62.5(4) | 37.5(4) |
| $Yb_5Sb_3H_{0.75}$ | 62.4(5) | 37.6(5) |
| $Yb_5Sb_3H_1$ | 62.8(9) | 37.8(9) |

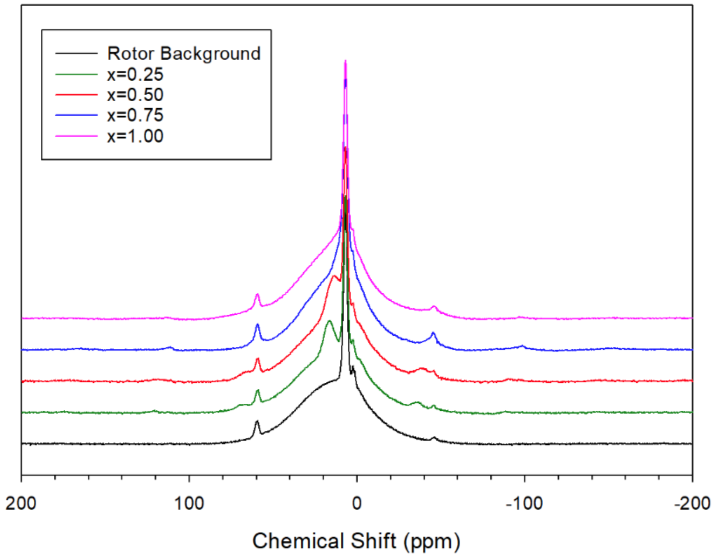


**Figure S4**. $^1H$ MAS NMR spectra of $Yb_5Sb_3H_x$ with $x$ = 0.25, 0.50, 0.75 and 1.0. Instrument $^1H$ background (show in black) is also displayed for comparison.

The $^1H$ MAS NMR spectrum from an empty rotor has a broad signal and a narrow signal with spinning sidebands. Using Bruker's Topspin software, and an NMR spectral fitting software DMFIT (https://nmr.cemhti.cnrs-orleans.fr/dmfit/) we simulated the experimental spectrum of the background signal with two components. The fitting parameters are given in Table S5.

The spectrum can be fitted with a relatively narrow signal with the isotropic chemical shift at 6.63 ppm with spinning sidebands, and a broad signal centered at 13.5 ppm. The sum of the two simulated components is in good agreement with the experimental data, with 95.6% overlapping, leaving the uncertainty to less than 5%. The narrow signal with spinning sidebands can be assigned to the $^1H$ signals from the Vespel cap and bottom-tip. The broad signal can be assigned to the hydrogen atoms present in the stator block. The $^1H$ MAS NMR spectra of $Yb_5Sb_3H_x$ ($x$ = 0.25, 0.5, 0.75, and 1.0) showed extra signal intensities on top of the instrument background. The deconvolution results are displayed in Table S5.

**Table S5**. $^1$H MAS NMR spectral fitting parameters

| Sample | δ(iso) ppm | δ(CSA) ppm | η(CSA) | LB (Hz) | Integral | Assignment |
|---|---|---|---|---|---|---|
| Background | 6.63 | -55.58 | 0.06 | 1172 | 7.084E+8 | Rotor |
| | 13.5 | 23.57 | 0.44 | 26289 | 2.894E+9 | Probe |
| $Yb_5Sb_3H_x$, $x$ = 1.0 | 9.7 | 74.8 | 0.007 | 8846 | 1.193E+10[a] | |
| $Yb_5Sb_3H_x$, $x$ = 0.75 | 10.0 | 78.7 | 0.008 | 6542 | 8.822E+09[a] | |
| $Yb_5Sb_3H_x$, $x$ = 0.5 | 14.2 | 80.6 | 0.004 | 5292 | 7.922E+09[a] | |
| $Yb_5Sb_3H_x$, $x$ = 0.25 | 16.6 | 77.7 | 0.006 | 4198 | 6.567E+09[a] | |

δ(iso): Isotropic chemical shift
δ(CSA): Chemical Shift Anisotropy, the unit is in ppm with a Larmer frequency of 500.03 MHz
η(CSA): Asymmetry parameter
LB: Full width at half height
[a]after background subtraction and sample weight normalization

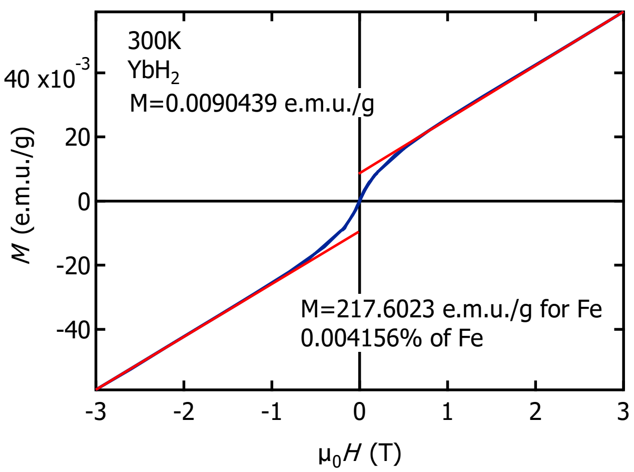


**Figure S5**. Magnetization measurements (at 300 K) for $YbH_2$ precursor. Calculations of the amount of Fe impurity that would contribute to the ferromagnetic component of the $Yb_5Sb_3H_x$ samples are shown.

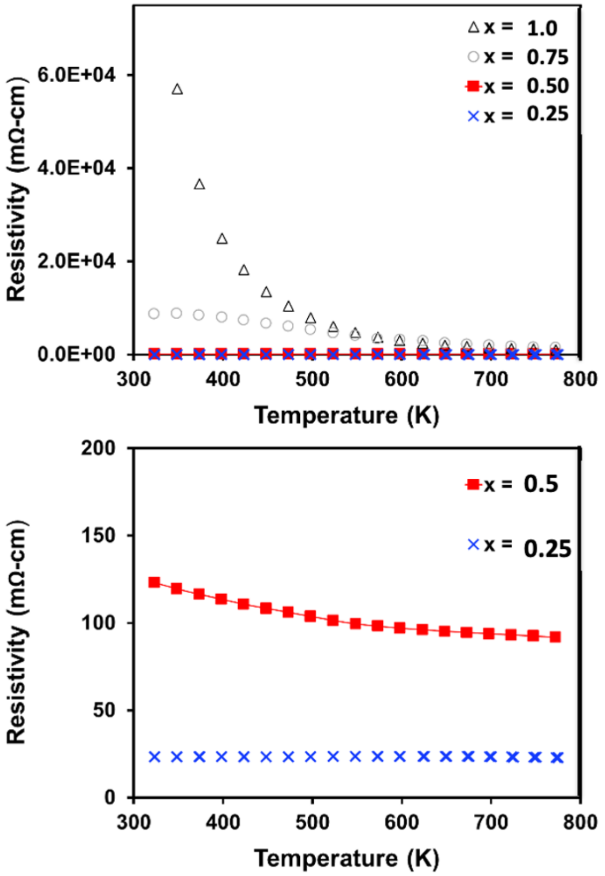


**Figure S6**. Experimental electrical resistivity data of $Yb_5Sb_3H_x$ with $x$ = 0.25, 0.50, 0.75 and 1.0 from the first heating cycle.

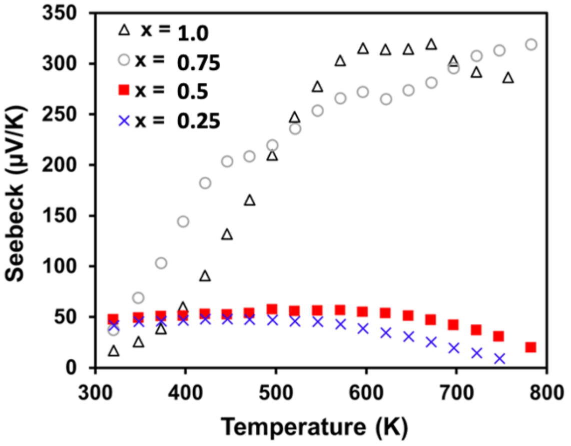


**Figure S7.** Experimental Seebeck data of $Yb_5Sb_3H_x$ with $x$ = 0.25, 0.50, 0.75 and 1.0 from the first heating cycle.

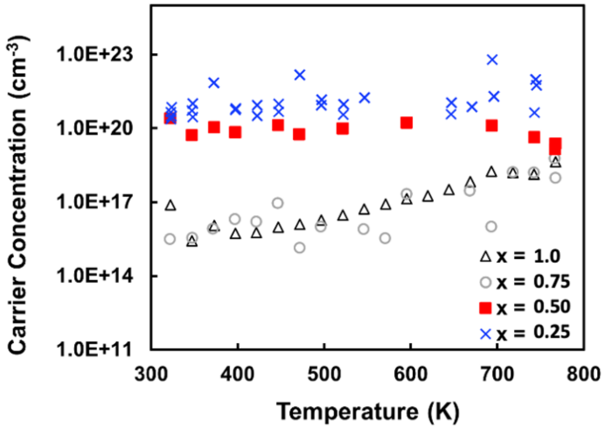


**Figure S8.** Experimental carrier concentration data of $Yb_5Sb_3H_x$ with $x$ = 0.25, 0.50, 0.75 and 1.0 from the first heating cycle.

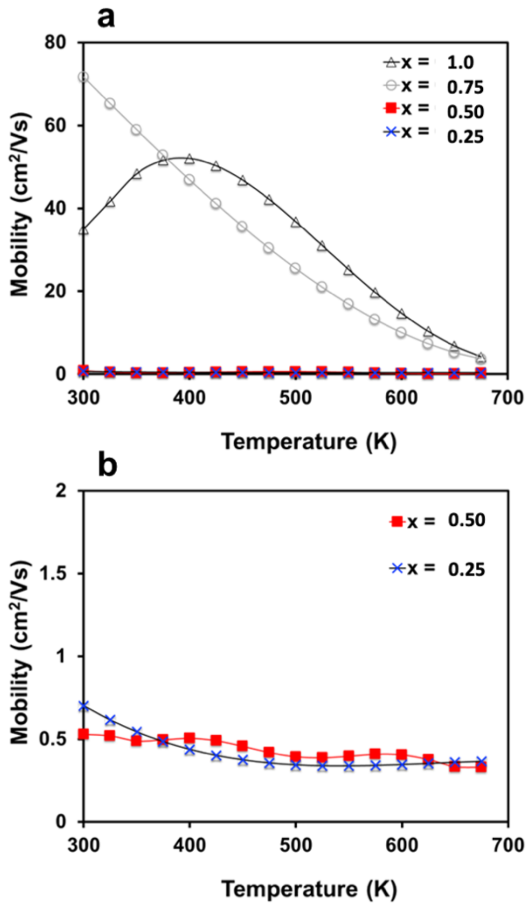


**Figure S9.** Experimental mobility data of $Yb_5Sb_3H_x$ with $x$ = 0.25, 0.50, 0.75 and 1.0 from the first heating cycle.

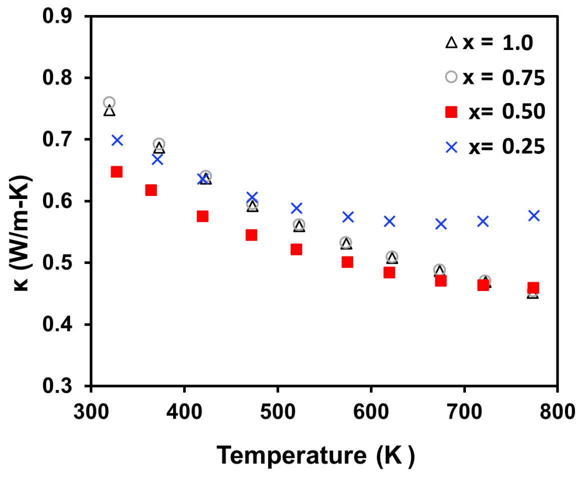


**Figure S10.** Experimental thermal conductivity data of $Yb_5Sb_3H_x$ with $x = 0.25$, 0.50, 0.75 and 1.0 from the first heating cycle.
